# Comparative analysis of iPSC-derived NK cells from two differentiation strategies reveals distinct signatures and cytotoxic activities

**DOI:** 10.3389/fimmu.2024.1463736

**Published:** 2024-10-09

**Authors:** Matthias Huyghe, Christophe Desterke, Jusuf Imeri, Nathan Belliard, Diana Chaker, Noufissa Oudrirhi, Hudson Bezerra, Ali G. Turhan, Annelise Bennaceur-Griscelli, Frank Griscelli

**Affiliations:** ^1^ Institut National de la Santé et de la Recherche Médicale (INSERM), Unité Mixte de Recherche en Santé (UMR-S-1310), Villejuif, France; ^2^ Unités Mixtes de Service (UMS 045)- CITHERA (Center for iPSC Cell Therapy), National Infrastructure INGESTEM, Corbeil-Essonnes, Evry, France; ^3^ Service d’Hématologie Biologique Unité d’Onco-Hématologie moléculaire et Cytogénétique Assistance Publique - Hôpitaux de Paris (APHP), Hôpital Universitaire Paris Sud Paul-Brousse, Villejuif, France; ^4^ Université Paris-Saclay, Faculté de Médecine, Kremlin Bicêtre, France; ^5^ Université Paris Cité, Faculté des Sciences Pharmaceutiques et Biologiques, Paris, France; ^6^ Institut Gustave-Roussy, Département de Biologie et Pathologie Médicale, Villejuif, France

**Keywords:** induced pluripotent stem cell (iPSC), natural killer (NK), immunotherapy, feeder-free differentiation, lymphoid-based differentiation

## Abstract

**Purpose:**

The ability to generate natural killer (NK) cells from induced pluripotent stem cells (iPSCs) has given rise to new possibilities for the large-scale production of homogeneous immunotherapeutic cellular products and opened new avenues towards the creation of “off-the-shelf” cancer immunotherapies. However, the differentiation of NK cells from iPSCs remains poorly understood, particularly regarding the ontogenic landscape of iPSC-derived NK (iNK) cells produced *in vitro* and the influence that the differentiation strategy employed may have on the iNK profile.

**Methods:**

To investigate this question, we conducted a comparative analysis of two sets of iNK cells generated from the same iPSC line using two different protocols: (i) a short-term, clinically compatible feeder-free protocol corresponding to primitive hematopoiesis, and (ii) a lymphoid-based protocol representing the definitive hematopoietic step.

**Results and discussion:**

Our work demonstrated that both protocols are capable of producing functional iNK cells. However, the two sets of resulting iNKs exhibited distinct phenotypes and transcriptomic profiles. The lymphoid-based differentiation approach generated iNKs with a more mature and activated profile, which demonstrated higher cytotoxicity against cancer cell lines compared to iNK cells produced under short-term feeder-free conditions suggesting that the differentiation strategy must be considered when designing iNK cell–based adoptive immunotherapies.

## 1 Introduction

Immune cell–based therapies have shown promise as salvage therapies for several types of malignancies refractory to conventional treatments. In this area, impressive progress has been made over the past decade with the use of T-infiltrating lymphocytes (TIL) in solid tumors and chimeric antigen receptor (CAR) T cells, which have been widely investigated in the treatment of lymphoid hematopoietic malignancies (1). However, autologous T-cell therapies suffer from significant interindividual variability, severe side effects, and very high costs. Because of this, there is a need to develop ready-to-use allogeneic immunotherapies for cancer, and in this context, the use NK cells is a promising approach (2).

NK cells are cytotoxic innate lymphoid cells that play a pivotal role in immune surveillance. They are capable of eliminating cancerous or virally infected cells via their stress signals. Unlike T cells, the cytotoxicity of NK cells is not limited by the target cell’s expression of molecules of the major histocompatibility complex (MHC), thus providing broader tumor recognition, avoiding the risk of graft-versus-host disease (GVHD), and allowing these cells to be used in allogeneic settings. NK cells can also modulate the adaptive response by secreting various chemokines and cytokines, such as interferon-gamma (IFN-γ) and tumor necrosis factor α (TNF-α) (3, 4). Various strategies have been developed, for the generation of large quantities of allogeneic NK cells from multiple sources, such as peripheral blood mononuclear cells (PBMCs), cord blood units, or iPSCs each with its own advantages and disadvantages (5). Donor-derived NK cells can be rapidly expanded to produce mature NK cells, but this process is often hindered by quantity limitations and inter-donor variability. In response to these challenges, iPSCs have emerged as an interesting source for NK cell-based therapy (6).

Embryonic and induced pluripotent stem cells represent an invaluable starting material for the generation of a potentially unlimited number of functional NK cells (7–11). Moreover, iPSCs can be readily genetically engineered to generate gene-edited NK cells that exhibit specific modifications to enhance their functionality (12) and/or the expression of fusion proteins like the CAR construct (13). The generation of iNK cells thus has enormous potential for use in developing off-the-shelf antitumor therapies.

NK cells develop within the bone marrow and secondary lymphoid organs in adults (14–16). Their differentiation and maturation proceed through five distinct stages. Stages 1 and 2 encompass early lymphoid-committed progenitors characterized by the expression of CD34^+^CD45RA^+^CD7^+^c-kit^+^. Stage 3 refers to the earliest committed precursors in the NK cell lineage, capable of differentiating exclusively into NK cells both *in vitro* and *in vivo*. From stage 4 onwards, NK cells express the CD56 marker along with other activating receptors essential for NK functionality, such as NKG2A, NKG2D, NKp46, and NKp30. Although stage 4 cells are functionally active, they can be further subdivided into stages 4a and 4b. Stage 4b is associated with functional maturity, marked by increased expression of the transcription factors T-BET and EOMES and the production of IFN-γ. Stage 4b is also characterized by the expression of the NKp80 marker. Subsequently, NK cells progress to stage 5, defined by the expression of CD16 and KIR markers. The process of NK cell differentiation remains a topic of ongoing debate (4, 14, 17–20).

The development of NK cells in embryos is also a subject of ongoing debate. NK cells first appear in the fetal liver around the 6^th^ week of development, coinciding with the migration of early hematopoietic cells to the fetal liver. While the precise origin of these NK cells remains unclear, evidence of progenitors in both the fetal liver and yolk sac suggests that NK cells and Innate lymphoid cells (ILCs) may originate from a multipotent progenitor that is not restricted to hematopoietic stem cells. Additionally, hematopoietic progenitors from the Aorta-Gonad-Mesonephros (AGM) and yolk sac, during the late 2^nd^ to 6^th^ weeks of development, have demonstrated the capacity to differentiate into NK cells (21–25).

Over the last decade, several approaches have been developed for differentiating NK cells from pluripotent stem cells (PSCs). Typically, this differentiation process involves an intermediate stage of hematopoietic progenitors (HPCs). Protocols for differentiating PSCs, including iPSCs, into HPCs are now well-established. Numerous protocols have been developed that can generate HPCs with varying degrees of maturity, most of which do not require stromal cells or animal-derived proteins (26, 27). The HPCs are then differentiated towards the NK cell lineage through the use of specific cytokines, notably IL7 and IL15, and/or mouse feeder cell lines (e.g., EL08, OP9, or MS5) (7–10). Feeder cell lines can be modified to enhance NK cell differentiation by expressing different Notch ligands, which are crucial for lymphoid induction (28–30), or HOXB4, which can enhance the proliferation and the cytotoxic activities of the NK cells (11, 31). Currently, OP9 cells expressing Notch ligands are the most commonly used cell line for producing NK cells (32–35). However, as feeder-based protocols are not appropriate for clinical purposes, recent years have seen the development of new clinically compatible protocols that aim to differentiate NK cells in defined conditions (36). The advantage of these methods, such as feeder-free protocols, includes a more robust manufacturing process due to the lack of animal cells and animal-derived serum, which reduces the risks of microbiological safety issues and variability, making them more suitable for clinical purposes. Yet, the feeder-free protocol presented by Zhu et al. relies on short term hematopoietic differentiation followed by NK cell differentiation in a feeder-free reduced environment, without artificial induction of the Notch signaling pathway.

To gain a better understanding of NK cell differentiation, we conducted a comparative analysis of iPSC-derived NK cells using both a feeder-free and a canonical feeder-based differentiation strategy. To minimize the interindividual and epigenetic variability inherent in iPSCs, we used the same iPSC line derived from peripheral blood mononuclear cells (PBMCs) to compare both protocols. Through the analysis of phenotypic characteristics, transcriptional profiles, and *in vitro* assays, we found that iNK cells generated via the feeder-based strategy exhibited enhanced activity and a more mature functional profile compared to those produced with the feeder-free protocol. This study suggests that although functional iNK cells can be obtained, the cells are not equivalent, and the influence of the differentiation strategy must be considered when designing cancer immunotherapies.

## 2 Materials and methods

### 2.1 Cell line

K562 cells were maintained in RPMI 1640 medium (Gibco, #1875093) supplemented with 10% fetal bovine serum (FBS) (Gibco, #26140079) and penicillin/streptomycin (Peni-Strep) (1%) (Gibco, #15140130) in a 5% CO_2_ incubator. OP9-DLL4 cells were maintained in α-MEM (Gibco, #12571063) supplemented with 20% FBS and penicillin/streptomycin (1%) in a 5% CO_2_ incubator. Anonymous human donor PBMCs were obtained by Ficoll centrifugation. NK cells were purified using RosetteSep (StemCell Technologies, #15025). The Lenti-X 293T cell line were cultivated according to Imeri et al., 2024 (37). Briefly, The Lenti-X 293T cell line was cultured in Dulbecco’s Modified Eagle’s Medium (DMEM) supplemented with 10% FBS.

### 2.2 iPSC culture

iPSC lines were generated by the Center for IPSC Therapies (CiTHERA, Genopole, Evry, France). The iPSC line used for this study (PB68) was derived from PBMCs of a healthy donor using the Sendai virus (ThermoFisher, #A16517). A Master Bank of one clone (PB68.6) was produced and qualified. Pluripotency was assessed by FACS analysis of the expression of the markers Oct4, Nanog, Tra1-60, and SSEA4. Pluripotency was also assessed by teratoma formation assay after intra-muscular injection of 3x10^6^ of iPSCs into 6-week-old NOD/SCID mice (Charles River Laboratories). After ten weeks, teratoma was dissected and fixed in 4% paraformaldehyde, embedded in paraffin and stained with hematoxylin and eosin in order to assess the presence of ectodermic, endodermic and mesodermic tissues. Genomic integrity was confirmed by conventional karyotype (46 XY). The PB68.6 line was cultured in Essential 8 Flex medium (Gibco, #A1517001) on Geltrex (Gibco, #A1413202). Passages were performed at confluence using 0.5 mM ethylenediaminetetraacetic acid (EDTA) (Invitrogen, #15575020) in phosphate-buffered saline (DPBS) solution (Gibco, #14190144).

### 2.3 NK differentiation protocols

For the feeder-free derivation of iNKs from iPSCs (feeder-free iNKs), cells were differentiated according to the protocol of H. Zhu et al., 2019 (36). Briefly, a single-cell suspension of iPSCs was seeded at 8000 cells per well in a 96-well plate containing APEL2 medium (Stemcell Tech, #78062) supplemented with cytokines (40 ng/mL SCF, Stemcell Tech, #78062; 20 ng/mL BMP-4, Stemcell Tech, #78211.1; and 20 ng/mL VEGF-165, Stemcell Tech, #78073.1) and 10 μM ROCKi (Y-27632) (Stemcell Tech, #72304). The plate was then centrifuged at 300g for 5 min and incubated at 37°C in 5% CO_2_ for 6 days. The resulting embryoid bodies (EBs) were transferred to a 6-well plate containing NK cell differentiation medium (56.6% DMEM and 28.3% F12 (Gibco, # 11765054), 15% heat-inactivated human AB serum (Sigma Aldrich, # H3667), 1% P/S, 1% GlutaMAXTM (Gibco, #35050038), 1 μM β-mercaptoethanol (Sigma Aldrich, # M3148), 1% ITS supplement (Sigma-Aldrich, I3146), 20 mg/L ascorbic acid (Sigma-Aldrich A4403)) supplemented with cytokines: 20 ng/mL SCF, 20 ng/mL IL-7 (Stemcell Tech, #78196.1), 10 ng/mL IL-15 (Stemcell Tech, #78031), 10 ng/m FLT3 ligand (FLT3L) (Stemcell Tech, #78009), and 5 ng/mL IL-3 (Stemcell Tech, #78040) (1st week) for 3 weeks.

In the feeder-based protocol, iPSC-derived iNK (OP9-DLL4 iNK) cells were differentiated using an adapted version of the protocol of Euchner et al., 2021 (32). iPSCs were detached using EDTA, transferred to low-attachment plates, and incubated overnight in APEL2 medium containing 10 µM ROCKi (Y-27632) to allow EB formation. EBs were collected the next day and transferred into APEL2 medium supplemented with 40 ng/mL BMP-4, 10 ng/mL bFGF (Stemcell Tech, #78188.1) and 50 ng/mL VEGF. On day 4, the EBs were collected again and transferred into APEL2 medium supplemented with 50 ng/mL SCF, 20 ng/mL Flt3L, 20 ng/mL IL-3, and 30 ng/mL TPO (Stemcell Tech, #78210.1). On day 14, EBs were collected and transferred onto overgrown OP9-DLL4 stromal cells in α-MEM supplemented with 20% FBS, 20 ng/mL SCF, 5 ng/mL FLT3l, 5 ng/mL IL-7, and 10 ng/mL IL-15. iNK cells were harvested and transferred every week onto new OP9-DLL4 cells.

### 2.4 Cytometry

For extracellular staining, cells were washed with DPBS and incubated with antibodies in a volume of 100 mL of DPBS with an adequate amount of antibody (**Supplemental Table 1**). For intracellular staining, cells were then fixed with BD Cytofix™ (BD, 554655) for 10 minutes at room temperature, protected from light, washed, and permeabilized with BD Perm/Wash™ (BD, 555028) together with intracellular antibodies for 20 minutes at 4°C. Flow cytometry was performed with a BD FACS LSRFortessa™ analyzer and Miltenyi MACSQuant 10™ data were examined using FlowJo software version 10.1 (Tree Star, Inc.).

### 2.5 Proliferation assay

NK cells were incubated in NK containing RPMI 1640 with 10% FBS (Gibco, #A5256701), 1% GlutaMAX™ Supplement (Gibco, #35050038), and 50 UI/mL of human IL-2 (Miltenyi, #130-097-743). NK cells were cultivated overnight and then harvested for intracellular staining of Ki67.

### 2.6 NK expansion assay

NK cells were tested for their expansion ability using artificial antigen presenting cells (aAPCs) K562-mbIL21. aAPCs were generated using lentivirus expression cassette containing membrane bound IL-21 fusion protein described by Denman et al., 2012 (38). In order to produce mbIL21 expressing lentiviruses, we used Lenti-X 293T as a packaging cell line and ps-PAX2.2, and pMD2.G as packaging vector and envelope vector, respectively. Briefly, the Lentix-293T cells were co-transfected by lipofectamine 3000 reagent (ThermoFisher, L3000015) with 20µg packaging vector of ps-PAX2.2 (Addgene), 10µg envelope vector of pMD2.G (Addgene), and 30µg of mbIL21 vector, synthetized by VectorBuilder. iNK were coculture at a ratio of 1∶2 (iNK∶aAPC) in NK expansion medium. Cultures were refreshed with half-volume media changes every two to three days, and re-stimulated with aAPCs at ratio of 1∶1 every seven days.

### 2.7 RNA sequencing

Primary NK cells were harvested by negative selection from healthy donor blood using RosetteSep™ Human NK Cell Enrichment Cocktail (Stemcell Tech, #15025). Total RNA was extracted using the RNeasy Mini Kit (Qiagen, #74104). A total RNA library was prepared with TrueSeq technology (Illumina) and paired-end reads of 75 pb were sequenced on an Illumina apparatus (Illumina). Fastq files were aligned using the STAR algorithm (version 2.7.6a) (39) with two-pass basic mode and the Ensembl reference genome release 101. Reads were then counted using RSEM (version 1.3.1) (40) and statistical analyses were performed on the read counts with the DESeq2 package (DESeq2_1.26.0) of R (version 3.6.3) using the standard DESeq2 normalization method (median of ratios) and a pre-filter of reads and genes (reads uniquely mapped on the genome, or up to 10 different loci with a count adjustment, and genes with at least 10 rads in at least 3 different samples) (41). Supervised differential gene expression analysis was performed with EdgeR version 3.40.2 to compare experimental conditions (42). Geneset enrichment analysis was performed with the GSEA standalone application (43) version 2-2.2.2.4, based on the Hallmarks MSigDb database version 2023 (44). Expression heatmaps were drawn with the Pheatmap R-package version 1.0.12.

### 2.8 Prediction of transcription factor interactions in the transcriptome

Based on the FANTOM consortium database of known interactions between human transcription factors (45), an R-package was designed to collect and test interactions between the transcription factors that were found to be differentially expressed in the human transcriptome. These analyses produce a combination score and an enrichment network depicting the combinations between transcription factors. This package is available at the following address: https://github.com/cdesterke/tfcombined (accessed on January 15th, 2024).

### 2.9 Developmental signature of NK cells

To characterize the developmental signature of NK cells, single-cell transcriptome data were obtained from an immune development cell atlas (https://developmental.cellatlas.io/fetal-immune) and used to analyze the genes that were differentially expressed between human NKs from fetal bone marrow and from yolk sac (24). The top 100 differentially expressed genes between NK cells in these developmental conditions were then employed to distinguish between transcriptome conditions from feeder-free iNKs and OP9-DLL4 iNKs. Volcano plots and expression heatmaps were created with the transpipe 1.4.0 R-package (https://github.com/cdesterke/transpipe14, accessed on April 2^nd^, 2024) following limma analysis (46).

### 2.10 Degranulation assay

NK cells stained with CD56-BV421 were incubated with or without cancer targets (K562 cells) at a 1:2 ratio of effector to target. CD107a-PEVio770 antibody was added to each well and incubated for 1 hour. Then, Monensin (BD GolgiStop™ Protein Transport Inhibitor, BD Biosciences, #554724) and Brefeldin A (BD GolgiPlug™ Protein Transport Inhibitor, BD Biosciences, #555029) were added and incubated for 2 more hours. CD107a expression was evaluated following data normalization of NK cells without target cell co-culture and a PMA/ionomycin positive control.

### 2.11 ELISA assay

NK cells were incubated overnight with or without cancer target cells (K562) at a 1:2 ratio of effector to target. Supernatants were tested according to the manufacturer’s instructions with the IFN-γ ELISA kit (ThermoFisher, #KAC1231) and the Granzyme B ELISA kit (ThermoFisher, #BMS2027-2). Results that were below the detection limits were considered to be 0 for statistical purposes.

### 2.12 Cytotoxicity assay

Cytotoxicity induced by NK cells was assessed by staining the surface of target cells first with CellTrace Yellow (ThermoFisher Scientific, #C34567) and then Annexin V. For CellTrace staining, cells were incubated with 1 μL of the dye per 10^6^ cells/mL in PBS for 20 min at 37°C. Stained target cells were then co-cultured at different ratios with iNK cells for 2 h and subsequently stained with Annexin V – APC (ThermoFisher Scientific, #88-8007-74) according to the manufacturer’s instructions. Briefly, cells were washed with 1X binding buffer, centrifuged, and resuspended in binding buffer containing Annexin V – APC. After 15 minutes of incubation, cells were washed again and analyzed by FACS.

### 2.13 Statistical analysis

Two-tailed Student’s t-tests were performed using GraphPad Prism (GraphPad Software, Boston, Massachusetts, USA). Means and SD are depicted on the graphs. All experiments were performed three times. ns, not significant; *, P < 0.05; **, P < 0.01; ***, P < 0.001; ****, P < 0.0001.

## 3 Results

### 3.1 Generation of hematopoietic progenitor cells with high NK potential using both lymphoid-based and feeder-free protocols.

**Figure 1:**
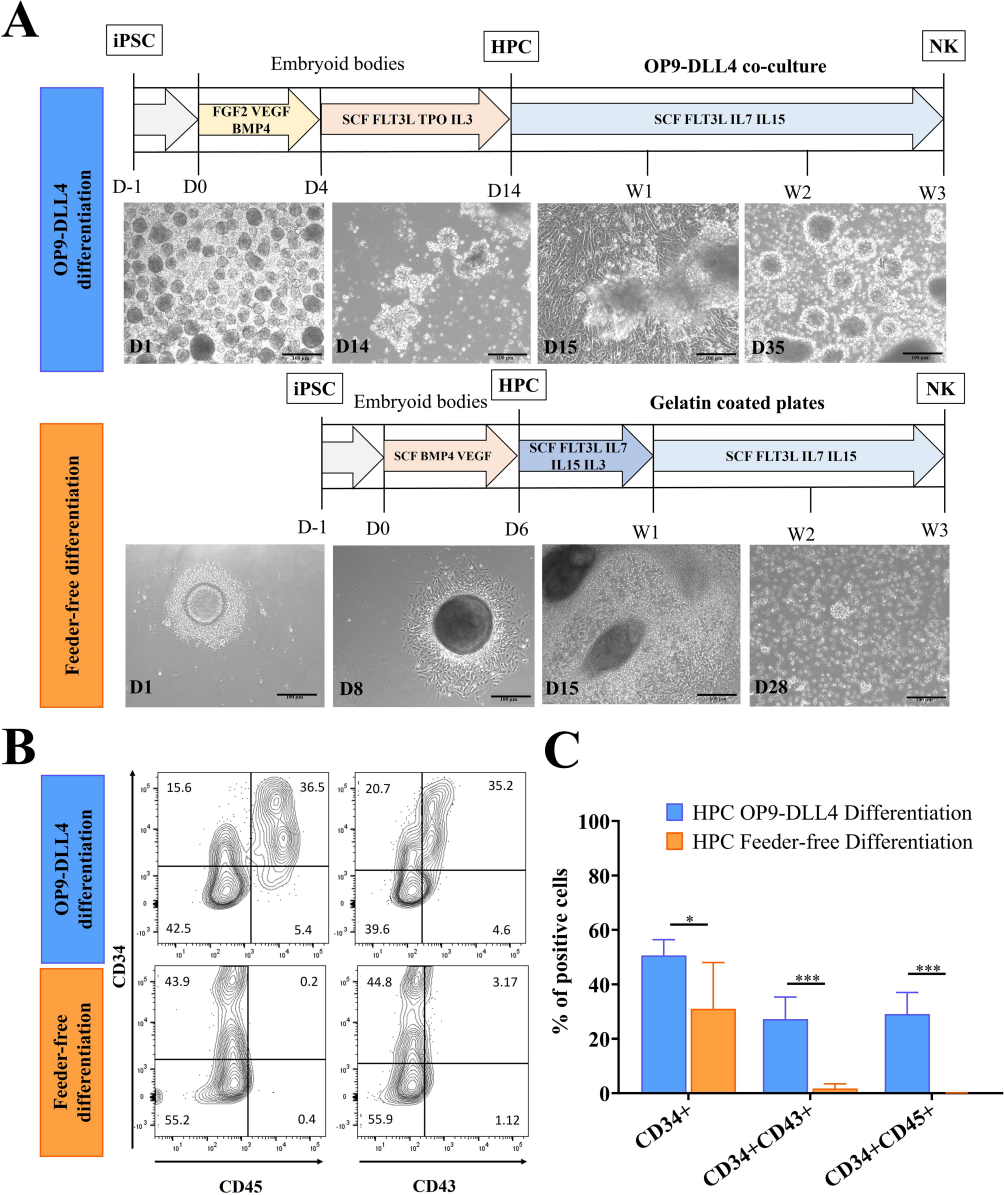
Generation of hematopoietic progenitor cells (HPCs) from iPSCs using two different protocols. **(A)** Outline of OP9-DLL4 differentiation protocol and feeder-free differentiation protocol; scale bar represents 100 µm. **(B)** Representative flow cytometry analysis of HPCs generated by the OP9-DLL4 protocol at day 14 and the Feeder-free protocol at day 6. **(C)** Phenotype of the generated HPCs. Means and SD are represented. Experiments were performed three times. P-values were calculated using a two-tailed Student’s t-test. *P < 0.05; ***P < 0.001.

All experiments were conducted using the same iPSC line (**Supplementary Figure 1**). The differentiation of hematopoietic progenitor cells (HPCs) was induced via two distinct methods: (i) the OP9-DLL4 protocol, in which 3D embryoid bodies (EBs) formed through self-aggregation in ultra-low-attachment (ULA) plates, and (ii) the feeder-free protocol, which employed spin aggregation of single-cell suspensions (**Figure 1A**). The profiles of the HPCs were then assessed at D14 (OP9-DLL4) or D6 (feeder-free) based on the expression of the CD34, CD45, and CD43 surface markers. Both protocols were able to produce CD34^+^ cells: 50.3 ± 5.8% for the OP9-DLL4 protocol and 27.9 ± 15.2% for the feeder-free protocol (P-value < 0.05). However, CD45 and CD43 were only found in cells derived using the OP9-DLL4 protocol (31.1 ± 6.1% CD34^+^CD45^+^ and 28.8 ± 7.1% CD34^+^CD43^+^; **Figures 1B, C**). Because enrichment of HPCs was not required, undigested D14 EBs (OP9-DLL4) and undigested D6 EBs (feeder-free) were then placed in NK differentiation conditions. To obtain a better understanding of the differentiation kinetics, we monitored the expression of CD34, CD45, and the NK lineage marker CD56 weekly. As expected, in both protocols, a decrease was observed during differentiation in the hematopoietic progenitor marker CD34 while expression of the pan-mature hematopoietic marker CD45 and the NK marker CD56 increased proportionally. We also monitored the emergence of lymphoid progenitors weekly throughout the differentiation process. A small population of [CD34^+^]CD7^+^CD45RA^+/-^ cells emerged 7 days after NK differentiation with the OP9-DLL4 protocol. This population appears to disappear by the second week of NK differentiation. However, we were unable to detect this population in the Feeder-free protocol. After 3 to 5 weeks of differentiation, almost 90% of floating cells in both protocols expressed CD56 (**Supplementary Figures 2A, B**).

### 3.2 Both protocols generate mature iNK cells, but with distinct phenotypes

**Figure 2:**
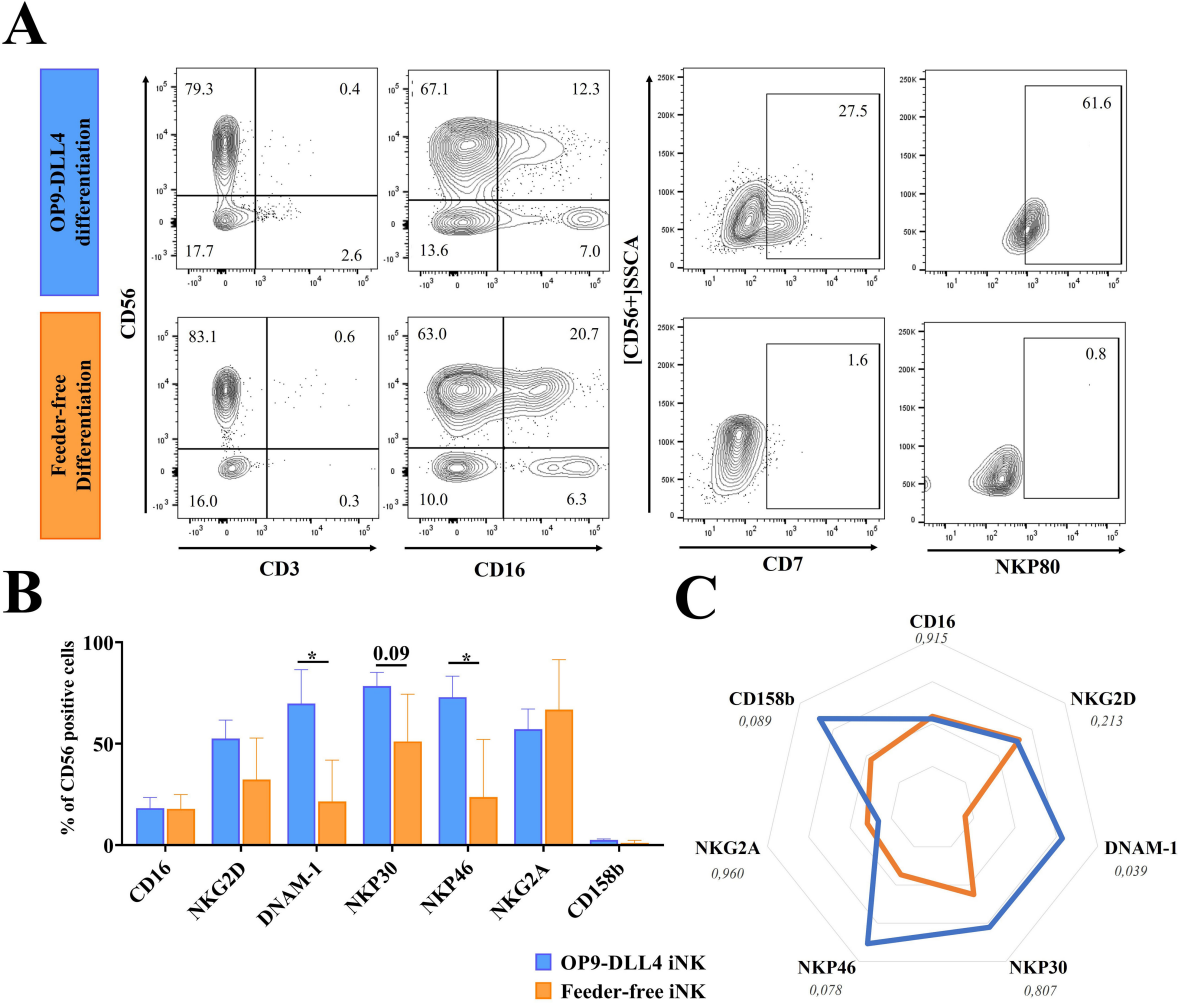
Generation of iNK cells. **(A)** Representative flow cytometry analysis of iNK cells generated by the OP9-DLL4 protocol and the feeder-free protocol after 3-5 weeks of culture in NK induction medium. **(B)** Phenotype of the generated iNK cells. **(C)** Relative mean fluorescence intensity (MFI) of CD107a expressing cells generated cells after 3-5 weeks in NK induction medium, from four independent differentiation cultures. Means and SD are represented. Experiments were performed three times. P-values were calculated using a two-tailed Student’s t-test. *P < 0.05.

The iNK cells generated from both protocols expressed the typical NK markers CD56^+^CD16^-^ and CD56^+^CD16^+^ and were negative for CD3 (**Figure 2A**). CD16 (FcγRIII) is responsible for the antibody-dependent cell-mediated cytotoxicity (ADCC) characteristic of NK cells. This marker is typically expressed in mature stage 5 blood-circulating NK cells in adults. Interestingly, it was found here to be expressed at similar levels in iNKs derived from both protocols (18.3 ± 9.1% for OP9-DLL4 iNKs and 17.9 ± 6.1% for feeder-free iNKs) in the CD56^+^ fraction (**Figure 2B**). Instead, CD7, which is a marker of lymphoid commitment, was found only in OP9-DLL4 iNKs; it appeared gradually in CD56^+^ cells at the endpoint of differentiation (27.5% in OP9-DLL4 iNKs and 1.6% in feeder-free iNKs cells) (**Supplementary Figure 2C**). NKp80, which characterizes stage 4b mature and cytotoxic NK cells, was also detectable in up to 61.6% of OP9-DLL4 iNKs, but was undetectable in terminally differentiated feeder-free iNKs (**Figure 2A**).

Compared to feeder-free cells, OP9-DLL4 iNKs displayed a distinct phenotypic profile, with CD56^+^ populations exhibiting a significantly higher proportion of cells positive for DNAM-1 (69.8 ± 28.9% for OP9-DLL4 iNK cells versus 21.5 ± 17.6% for feeder-free iNK cells, P-value < 0.05) and NKp46 (72.9 ± 17.9% for OP9-DLL4 iNK cells versus 23.7 ± 24.6% for feeder-free iNK cells, P-value < 0.05). Instead, the expression of the receptors NKP30, NKG2A, and NKG2D was similar in both protocols. Both sets of iNK cells also expressed the KIR receptor 2DL2-3 (CD158b) at low levels (2.6 ± 0.7% for OP9-DLL4 and 1.1 ± 1.1% for feeder-free) (**Figure 2B**). To better assess the profile of the CD56^+^ cell population at the endpoint of iNK differentiation, we looked at the relative expression of markers as measured by mean fluorescence intensity (MFI) (**Figure 2C**). The feeder-free iNK cells exhibited a generally muted expression profile compared to OP9-DLL4 iNKs, with a reduced expression of DNAM-1 (P-value < 0.05) and a relatively lower (though not significantly so) expression of NKP46 (P-value < 0.08).

To characterize their proliferation capacity, we stimulated iNK cells overnight with IL-2 (50 UI/mL) and analyzed the proportion of Ki67^+^ cells. The proportion of CD56^+^Ki67^+^cells was higher in OP9-DLL4 iNKs (33.1 ± 1.0%, compared to 8.8 ± 0.6% for feeder-free iNK cells), suggesting that they have an enhanced proliferation capacity (**Supplementary Figure 2D**). Taken together, these results suggest that, compared to feeder-free iNKs, OP9-DLL4 iNK cells exhibit a more differentiated, lymphoid-primed phenotype and express higher levels of activation receptors such as NKP46 and DNAM-1.

### 3.3 OP9-DLL4 iNK cells are enriched in inflammatory markers and transcription factors implicated in NK maturation and differentiation

**Figure 3:**
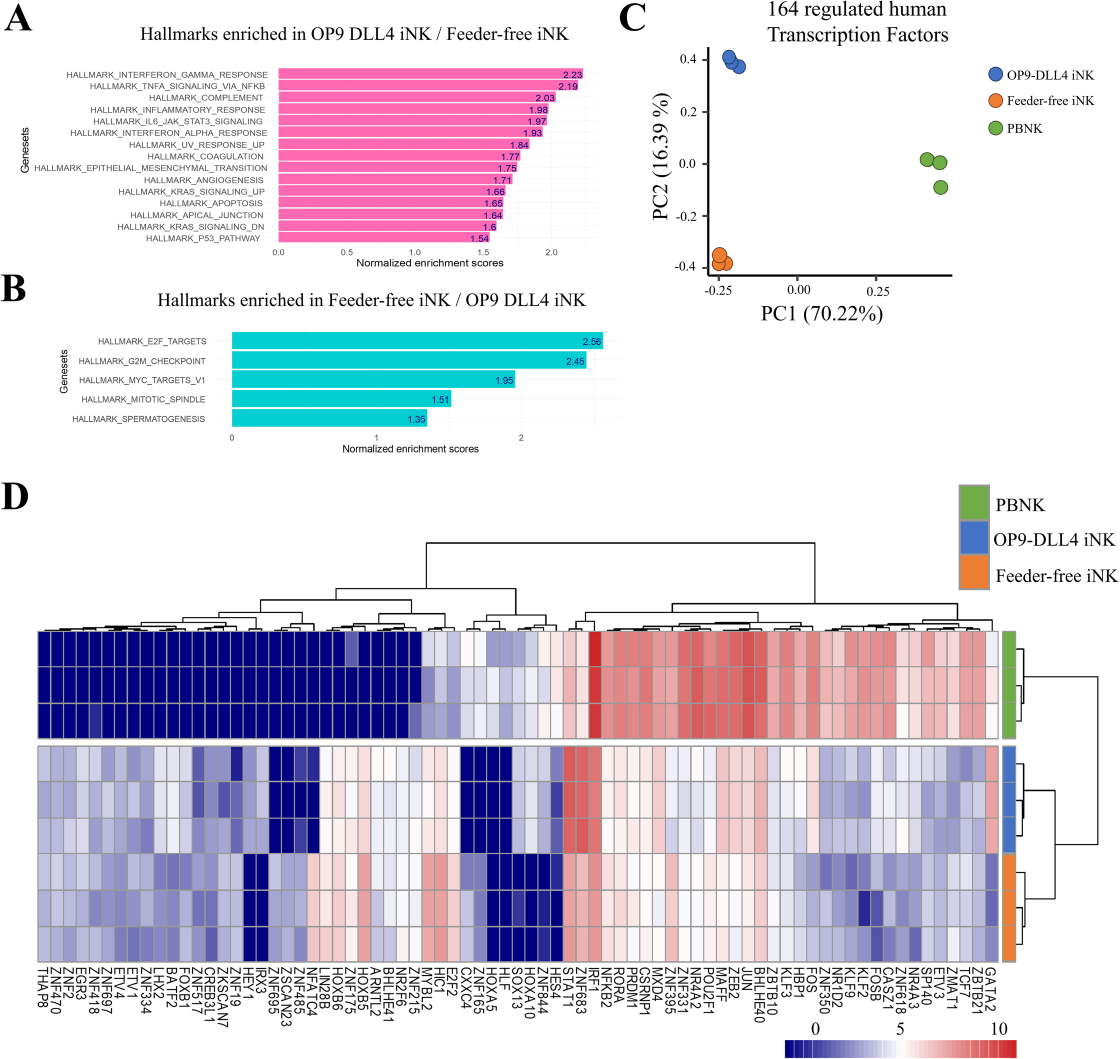
Transcriptional profile of generated iNK cells. **(A)** Barplot of normalized enrichment scores for hallmarks that were enriched in OP9-DLL4 iNKs relative to feeder-free iNKs. **(B)** Barplot of normalized enrichment scores for hallmarks that were enriched in feeder-free iNKs relative to OP9-DLL4 iNKs. **(C)** Principal component analysis (PCA) performed on the 164 human transcription factors (TF) with differential expression between iNKs and peripheral blood NK cells (PBNKs). **(D)** Expression heatmap of the 75 human transcription factors with the most significant differences in expression between experimental conditions (unsupervised clustering based on Euclidean distances).

To examine the generated NK cells in greater detail, we conducted a transcriptomic analysis of both OP9-DLL4 and feeder-free iNKs. Between these two sets of cells, we identified 2148 genes with significant differences in expression (differentially expressed genes, DEGs) (**Supplementary Figure 3**). Among these, we identified chemotactic markers such as *CCL17* and *CCL1*, which were upregulated in OP9-DLL4 iNK cells, as well as transcription factors like *GATA2.* We also detected the transcription factor *NFATC4* and the immune checkpoint *LAG3* as having higher expression in feeder-free iNK cells. Using the normalized transcriptome data, we conducted a gene set enrichment analysis (GSEA) comparing the two distinct sets of iNK cells using the Hallmark gene set database. OP9-DLL4 iNK cells showed significant enrichment in immune-related gene sets, including those involved in the IFN-γ response, TNF signaling via NF-κB, complement pathways, and inflammatory responses (**Figure 3A**). In contrast, GSEA revealed that feeder-free iNK cells exhibited enrichment in gene sets related to transcriptional regulation and cell cycle progression (**Figure 3B**).

The transcriptomes of our iNKs were also compared with those of peripheral blood NKs cells. Among the genes that were differentially expressed between the three conditions, we identified 164 human transcription factors (**Supplementary Table 1**). Moreover, unsupervised principal component analysis of the expression patterns of these 164 transcription factors was able to effectively discriminate among the three groups of cells: 70.22% of the variance distinguished primary NK cells from iNK cells along the first principal axis. In contrast, 16.39% separated OP9-DLL4 iNKs from feeder-free iNKs along the second principal axis (**Figure 3C**).

This pattern of segregation was further confirmed by unsupervised clustering based on the top 75 differentially regulated transcription factors (**Figure 3D**). Notably, OP9-DLL4 iNK cells exhibited major upregulation of *GATA2*, *FOS, FOSB, JUN, ZEB2*, and *STAT1* compared to feeder-free iNKs, while feeder-free iNK cells showed increased expression of *HOXB5*, *MYBL2*, *HC1*, and *E2F2* compared to OP9-DLL4 iNKs. Additionally, analysis of the transcription factors highlighted in OP9-DLL4 iNK cells revealed the enrichment of a comprehensive cistrome comprising interactions among *IRF1*, *FOS*, *STAT1*, *STAT2*, *STAT3*, *HES1*, and *GATA2*, all of which are known to be involved in NK cell differentiation and maturation. In contrast, feeder-free iNK cells were characterized by a major cistrome enriched in *MCM3*, *MCM7*, *MCM6*, and *MCM8*, which are implicated in replication and mitosis (**Supplementary Figure 4**).

### 3.4 iNK cells exhibit distinct developmental programs

**Figure 4:**
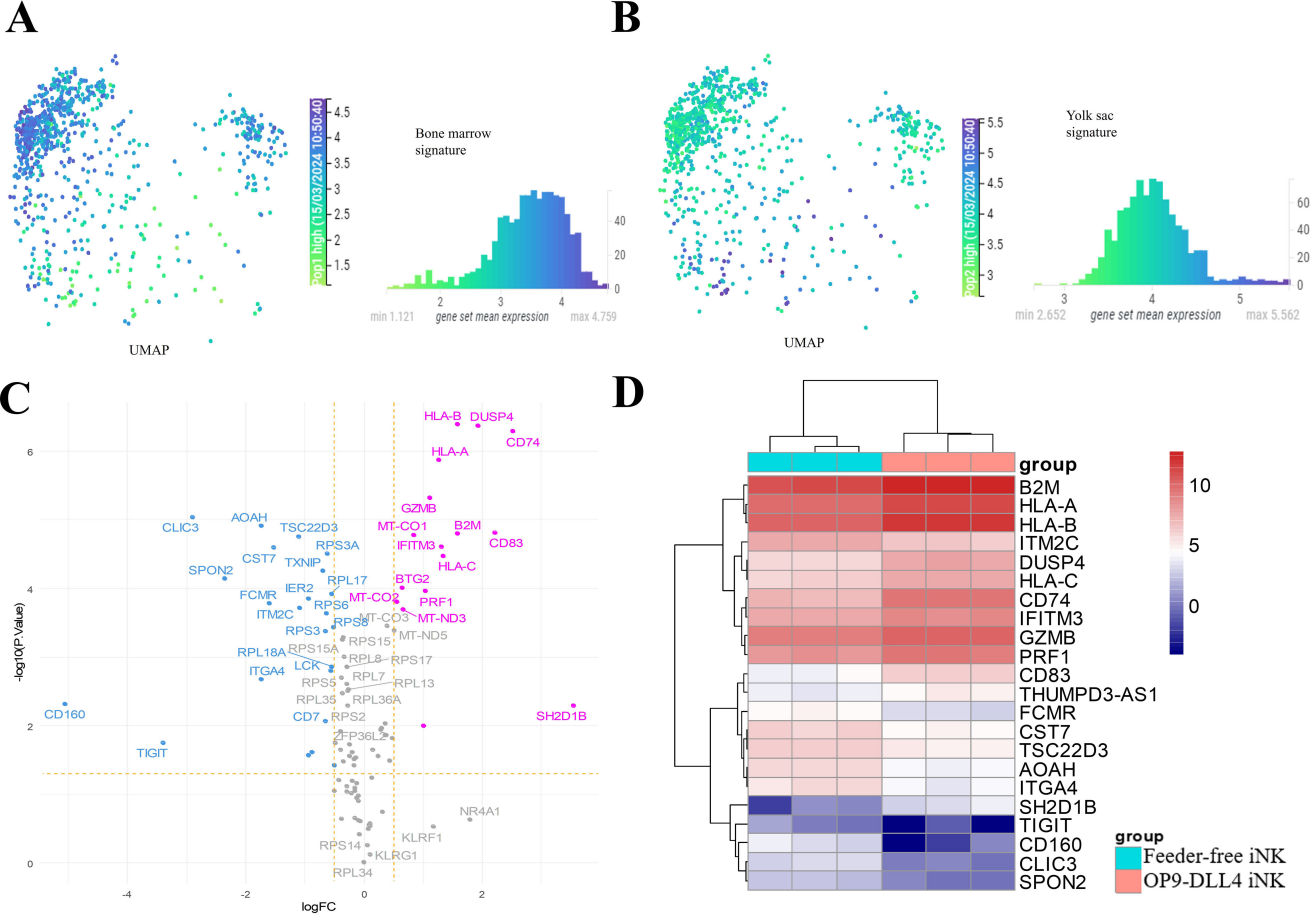
Regulatory differences in the human NK developmental program between iNK populations. **(A)** Single-cell UMAP dimension reduction and histogram of mean expression for the 50 best markers of human NK from fetal bone marrow. **(B)** Single-cell UMAP dimension reduction and histogram of mean expression for the 50 best markers of human NK from yolk sac. **(C)** Volcano plot of developmental NK genes with differences in expression between the two sets of iNKs created in this study (pink: overexpressed in OP9-DLL4 protocol; blue: overexpressed in feeder-free protocol). **(D)** Expression heatmap (clustering with Euclidean distances) of NK developmental genes with significant differences in expression between the two sets of iNK cells (logFC >=|1| and FDR P-value <=0.05).

To assess the generated iNK cells in an embryonic context, we analyzed the profile of NK cells derived from different anatomical sites using an immune development cell atlas (https://developmental.cellatlas.io/fetal-immune) (24). Specifically, we compared single-cell transcriptome data from the human yolk sac and fetal bone marrow using UMAP dimension reduction. This analysis revealed significant differences in gene expression profiles between these two cell populations. Effectively, after dimension reduction, bone marrow NK cells were found to be concentrated at the top left of the UMAP visualization of the human NK cell cluster (**Figure 4A**) and yolk sac NKs were found to be concentrated at the bottom of the heatmap (**Figure 4B**). Differential expression analysis was performed between the two NK identities and the top 50 differentially expressed genes (**Supplementary Table 2**) were identified in each cell population (histograms in **Figures 4A, B**). This NK developmental signature (100 genes) was then employed to distinguish between the iNK cell transcriptomes (**Figures 4C, D**). OP9-DLL4 iNK cells were found to be more enriched in *HLA*, *B2M*, *CD74*, *GRZB*, and *PRF1*, which are characteristic of fetal bone marrow NK cells. These results suggest that, compared to feeder-free iNKs, OP9-DLL4 iNK cells present a transcriptional profile closer to that of bone marrow NK cells.

### 3.5 OP9-DLL4 iNKs exhibit superior activity against the K562 cell line

**Figure 5:**
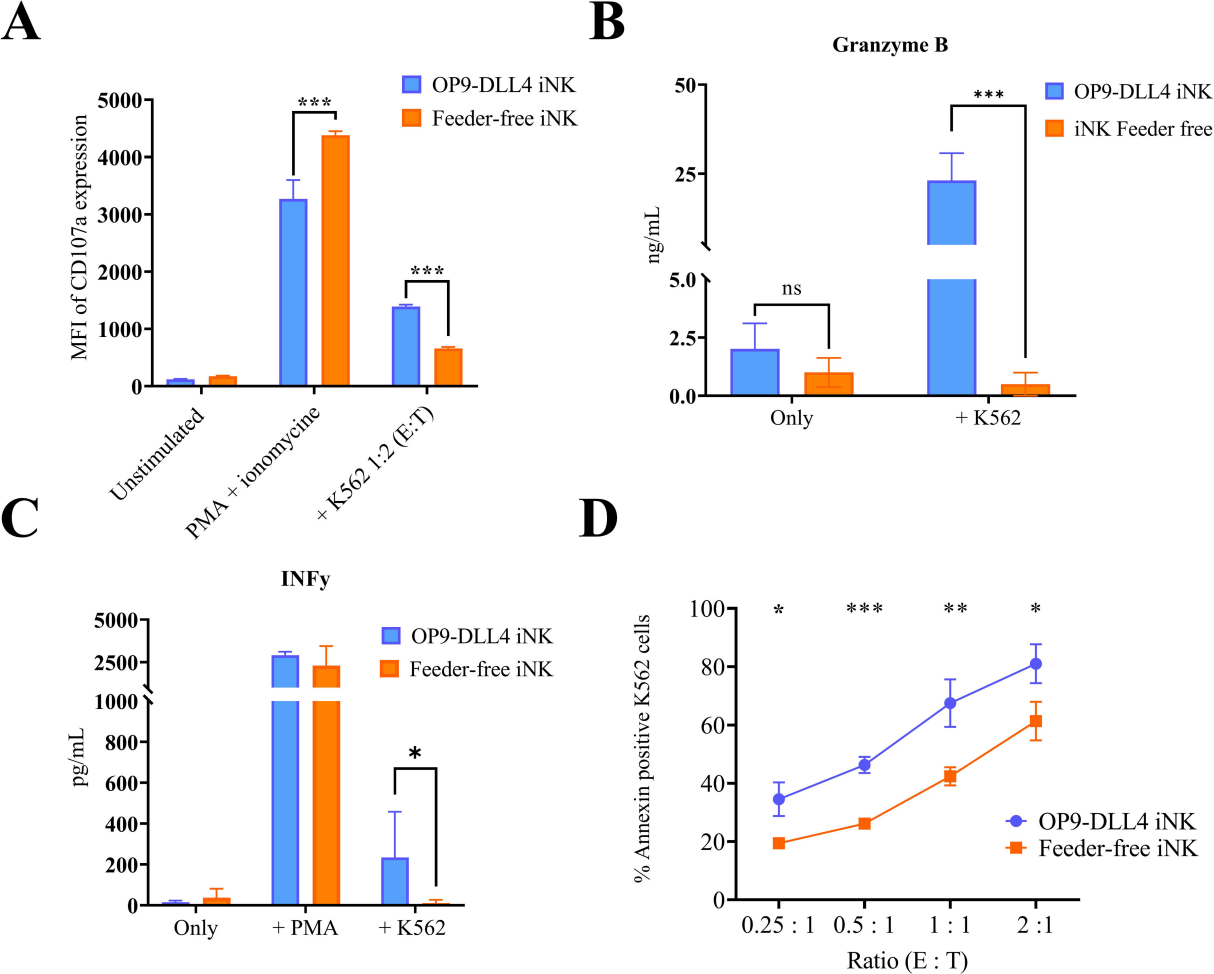
Degranulation and cytotoxicity studies on generated iNK cells. **(A)** Degranulation capacity of generated iNK cells assessed by CD107a expression after 3 h of stimulation with PMA or K562 cells. **(B)** Degranulation of Granzyme B by iNK cells after stimulation with K562, as assessed by ELISA. **(C)** Cytotoxic capacity of generated iNK cells as assessed by annexin expression on K562 cells after 4 h of coculture at different ratios of effector to target (E:T). **(D)** Secretion of IFN-γ after stimulation with K562, as assessed by ELISA. Means and SD are represented. Experiments were performed three times. P-values were calculated using a two-tailed Student’s t-test. ns, not significant; *P < 0.05; **P < 0.01; ***P < 0.001.

To assess the functionality of the iNK cells generated using feeder-free and feeder-based protocols, we investigated the degranulation capacity of these cells after co-culture with leukemic K562. As expected, iNK cells from both protocols were able to efficiently degranulate (more than 80% of cells in the positive control after PMA stimulation) (data not shown). After stimulation with K562 cells, OP9-DLL4 iNK cells showed a much higher degree of degranulation (1388.7 ± 35.2 MFI versus 657.3 ± 29.2 MFI for feeder-free iNKs, P-value < 0.0001) (**Figure 5A**). We corroborated this result by measuring, by ELISA assay, the concentration of granzyme B in the supernatant of overnight-stimulated iNK cells. As expected, OP9-DLL4 iNK cells showed a much higher concentration of granzyme B in the supernatant than feeder-free iNKs (23.1 ± 0.5 ng/mL and 0.5 ± 0.4 ng/mL, respectively, P-value < 0.0001) (**Figure 5B**). Similarly, we used ELISA assay to measure IFN-γ secretion in the supernatant after overnight stimulation with either PMA or K562 cells. Both types of iNK cells displayed a strong IFN-γ response to stimulation with PMA (2914.4 ± 140.0 pg/mL for OP9-DLL4 iNKs and 1929.9 ± 1104.3 pg/mL for feeder-free iNKs). However, IFN-γ secretion was much higher in OP9-DLL4 iNKs than in feeder-free iNKs after stimulation with K562 cells (234.1 ± 193.9 pg/ml versus 0.1 ± 0.03 pg/mL, respectively, P-value < 0.05) (**Figure 5C**). Finally, to evaluate the short term cytotoxic potential of these iNK cells, we evaluated the proportion of apoptotic cells resulting from co-culture with K562 cells at different ratios of effector to target (0.25:1, 0.5:1, 1:1, and 2:1). Interestingly, we observed a much higher percentage of apoptotic cells in all ratios with the OP9-DLL4 iNK co-culture (**Figure 5D**). Altogether, our data suggest that OP9-DLL4 iNKs are superior in terms of functionality in *in vitro* assays against the K562 cancerous cell line.

To further evaluate the cytotoxic potential of iNK cells, we generated artificial antigen-presenting cells (aAPCs) expressing mbIL21 (K562-mbIL21) to promote their expansion and maturation (**Supplementary Figure 5A**). This cell line appeared to activate iNK cells, evidenced by an increased expression of certain activating receptors such as DNAM-1 and NKp46, particularly in feeder-free iNK cells where expression levels were initially low (**Supplementary Figure 5B**). However, this cell line did not induce logarithmic expansion of iNK cells. After two weeks of co-culture, the cells exhibited unfortunately apoptotic morphology and showed signs of anergy in cytotoxicity assays against K562 cells (**Supplementary Figure 5C**).

## 4 Discussion

iPSC-derived NK cells have emerged as a promising resource for the development of “off-the-shelf” immunotherapies, demonstrating an *in vitro* efficacy comparable, or even superior, to that of PBMC-derived NK cells against various tumor cell lines (35). However, iNK cell-based therapies, similar to NK-based therapies from other sources, face significant challenges, including issues related to their *in vivo* persistence, response durability, tumor targeting, and tumor microenvironment (6). Additionally, the mechanisms underlying the differentiation of NK cells from iPSCs regarding the influence of the differentiation strategy on the iNK profile remain poorly understood.

We report here that two different differentiation strategies generated two sets of phenotypically and functionally distinct iNK cells, although both protocols were applied to the same iPSC line in order to minimize variability related to iPSC origin, clone, and epigenetic factors (47). Specifically, we compared a canonical lymphoid-based protocol based on OP9 cells that have been genetically engineered to express the Notch ligand DLL4 to promote HPC differentiation into NK cells (9, 32, 35) and a well-established feeder-free protocol used by several research teams (36, 48). To our knowledge, this is the first time this kind of work—a molecular and phenotypic comparative analysis of feeder-based and feeder-free differentiation strategies on iNK cells from the same iPSC clone—has been performed.

Previous research has documented how different differentiation strategies affect NK phenotype, maturity, and yield (8, 30, 33). Generally, HPCs can be induced towards the lymphoid lineage using different stromal cell lines (MS5, AFT024, EL08-1D2, OP9), with variable efficiency (7, 8, 49). In feeder-free differentiation, the strategy includes the formation of embryoid bodies using an autologous stroma to facilitate NK cell differentiation, although these hPSC-derived feeders are less effective than those derived using mouse feeder cells (8). The OP9 bone marrow–derived stromal cells are generally used for the generation of lymphoid-committed cells (50), particularly following genetic modification with the Notch ligand (28), and has been shown to drive hematopoietic cell maturation, thus enhancing the production of NK cells (30, 51).

It also has been demonstrated that different iNK cell profiles can be obtained *in vitro* from various HPCs, with particularly notable differences between Wnt-independent and Wnt-dependent HPCs (yolk sac erythromyeloid progenitor–like cells and pre-hematopoietic stem cells, respectively), as well as myeloid progenitors (52). Interestingly, NK cells derived from HSC-independent progenitors exhibit enhanced degranulation and direct cytotoxicity against cancerous targets. In contrast, NK cells derived from intra embryonic HSC-like progenitors demonstrate reduced degranulation and cytotoxicity but possess an enhanced ability to secrete cytokines, particularly IFN-γ. These observations support the hypothesis that NK cells differentiated from iPSCs may be functionally superior to those derived from CD34+ cells from cord blood or lymphoid progenitors (33, 53). It is important to emphasize that the NK cells were differentiated using either the OP9-DLL4 cell line or the fetal liver-derived AFT024 cell line.

The two differentiation protocols used here—OP9-DLL4 and feeder-free—are based on different strategies. Both require the formation of embryoid bodies (EBs) for the generation of HPCs, but they differ in terms of the timing and the types of cytokines used. Previous research has demonstrated how different protocols yield phenotypically different HPCs (26). With the OP9-DLL4 protocol, the resulting HPCs are largely CD34^+^CD45^+^, indicating a committed progenitor (26); the cells also express CD43, a common marker of hematopoietic induction (54). In contrast, HPCs generated from feeder-free protocols express only CD34, thus resembling an earlier, more primitive stage of hematopoietic progenitor (55). Similar results have been observed by multiple teams (36, 48). In the present study, EBs were transferred onto an OP9-DLL4 stromal layer for NK induction (feeder-based protocol) or placed directly in a gelatin-coated plate to let them form their own stroma to support NK development. Both sets of HPCs efficiently differentiated into NK cells, as evidenced by the progressive appearance of the CD45 and CD56 markers.

Both differentiation protocols were able to efficiently generate CD56^+^CD3^-^ iNK cells, although only a small proportion of iNKs were CD16^+^, which is consistent with the literature (8, 9, 32, 35, 48). Interestingly, the presence of the lymphoid marker CD7 was detected exclusively on OP9-DLL4 iNK cells. Adult NK cells express this marker from the common lymphoid progenitor CD34^+^CD7^+^CD45RA^+^CD10^-^ (21). However, feeder-free iNKs do not express CD7 at any point in the differentiation process. The exact role of CD7 in differentiation remains to be elicited, but these results suggest either that CD7 can be induced by the OP9-DLL4 stromal cell line or that CD7^+^ iNK cells have a differential lymphoid origin. To address this question, we monitored the emergence of common lymphoid progenitors marked by CD34^+^CD7^+^CD45RA^+^ through weekly analysis. A small population of CD34^+^CD7^+^CD45RA^+/-^ cells was detected during the OP9-DLL4 differentiation process. The expression of these markers appears transient and diminishes rapidly as more committed progenitors develop. Further analysis is necessary to evaluate the potential of these progenitors and their contributions to the OP9-DLL4 differentiation process. However, no CD34^+^CD45RA^+^ or CD34^+^CD7^+^ cells were observed during feeder-free differentiation, leaving the origin of lymphoid progenitors in this method undetermined.

Overall, OP9-DLL4 iNKs appeared to be more mature than feeder-free iNKs, as shown by the expression of the NKp80 marker in the former but not the latter. NKp80 is a critical marker defining functionally mature NK cells in adults (56). The two sets of iNK cells also displayed distinct phenotypes with respect to NK cell receptors, as evidenced by the differential expression of NKp30 and NKp46. Likewise, OP9-DLL4 iNKs expressed higher levels of DNAM-1, which is a critical marker for both NK and T cells for tumor immune surveillance and seems to be involved in the NK education process (57, 58). This result is also consistent with a more mature profile for OP9-DLL4 iNKs as NK cells acquire functional receptors through their differentiation (20). Both iNK populations were mostly KIR-negative, as previously reported in the literature (17, 35).

To further characterize both sets of generated iNKs, we performed transcriptomic analysis at the endpoint of differentiation. Compared to the feeder-free iNK cells, OP9-DLL4 iNKs were shown to be enriched in inflammatory markers, which is consistent with the more mature and activated phenotype of these cells. At the end of the differentiation process, OP9-DLL4 iNK cells induced the lysis of the OP9-DLL4 cells, and they may have also secreted IFN-γ, which could have contributed to the more-enriched inflammatory phenotype. However, no differences were observed between the iNKs in terms of basal secretions of IFN-γ. Conversely, feeder-free iNK cells were enriched in mitotic and proliferation pathways; interestingly, though, the proliferation assay revealed a more proliferative phenotype in OP9 DLL4 iNK cells.

Our analysis of differential gene expression highlighted the transcription factors with the most significant changes in expression between the two sets of iNKs as well as in PBNKs from different donors. As expected, PBNKs were enriched in NK cell transcription factors, with no substantial differences detected in the major NK TFs such as *ETS1*, *EOMES*, or *TBET* (20). However, certain transcription factors involved in NK cell maturation and differentiation were notably upregulated in the OP9-DLL4 iNK cell condition. For instance, ZEB2, a transcription factor essential for NK cell maturation and a key player in tumor cytotoxicity, showed increased expression (59, 60). Similarly, *STAT1* was also upregulated in OP9-DLL4 iNK cells (61).We also observed that OP9-DLL4 iNK cells exhibited higher expression of the FOS, FOSB, and JUN genes compared to feeder-free iNK cells. These transcription factors are known to form heterodimers, constituting the Activator Protein-1 (AP-1) transcription factor family, which is implicated in NK cell cytotoxicity (62, 63). The FOS-JUN heterodimer also appears to play a role in shaping NK cell chromatin towards an adaptive NK phenotype, potentially driving this process (64). OP9-DLL4 iNKs and Feeder-free iNKs exhibit also significant differences in the expression of *GATA2*, a transcription factor crucial for the development of NK and innate lymphoid cells (ILCs) in adults (65). In feeder-free iNKs, *GATA2* is expressed only at low levels. Interestingly, *GATA2* appears to form a cistrome with the transcription factor HES1, STAT3, STAT1, and FOS. Notably, both *HES1* and *GATA2* are regulated by the Notch signaling pathway (66–68). The regulation of this axis may provide insights into the differences in maturation and activity of generated iNK cells, potentially driven by the stimulation of the Notch pathway via the DLL4 ligand expressed by OP9 cells.

In order to better discriminate between the two populations of iNK cells, we compared them to developmental NK cell signatures from single-cell data of fetal bone marrow NK cells and yolk sac NK cells, obtained from an online immune development atlas published by Goh et al. (24). A differential expression analysis revealed interesting features, with a potentially deeper level of similarity between OP9-DLL4 iNK cells and bone marrow–derived fetal NK cells. The similarity between OP9-DLL4 iNK cells and fetal bone marrow NK cells was anticipated, as the OP9-DLL4 differentiation strategy relies on long-term hematopoietic differentiation and commitment to the lymphoid lineage within a bone marrow-derived stromal environment. This strategy is designed to closely mimic the natural ontogeny of NK cells during adult development. Based on the differentiation strategy, as well as the kinetic of the differentiation and the transcriptional profile analyses of Feeder-free iNK cells, we hypothesized that Feeder-free iNK cells might be ontogenically distinct, resembling NK cells derived from primitive extra-embryonic waves of hematopoiesis. It is known that primitive hematopoiesis, notably present in the yolk sac, exhibits strong NK potential (22, 33). While the ontogeny of feeder-free iNK cells was explored using single-cell data from fetal NK cells, the results remain inconclusive; however, they do highlight a distinct developmental program. Future analyses, such as single-cell transcriptomic comparisons at various time points during differentiation, could provide deeper insights into the exact ontogenic differences between the various iNK cells.

Finally, through various *in vitro* experiments, we confirmed that the superior phenotypic maturation and transcriptional profile of OP9-DLL4 translates into functional superiority in the secretion of IFN-γ and short-term cytotoxicity versus K562 cell line. Indeed, feeder-free iNK cells, albeit functional, were notably inferior to OP9-DLL4 iNKs in short term, degranulation and short term cytotoxicity, and IFN-γ secretion when co-culture with K562 cell line. K562 is the standard cell line primarily due to the downregulation of class I MHC, making it a preferential target for NK cells. Additional cytotoxicity studies, such as long-term cytotoxicity assays and serial killing assays of iNK cells, would provide valuable data to further dissect the functional differences, particularly when using other cancerous cell lines.

Following differentiation, iNK cells can be expanded to clinically relevant numbers using various techniques, including the use of aAPCs (38, 69) or cytokine stimulation to obtain memory-like NK cells (CIML) (70). These methods are well established for inducing metabolic and functional reprogramming of NK cells, thereby enhancing their cytotoxic potential (70). We conducted these experiments at the endpoint of the differentiation process with functional CD56^+^CD16^+/-^ iNK cells. However, it is possible to include an expansion step in the iNK cell production process. We attempted to develop an aAPC line in our laboratory based on the K562 mbIL21 model described by Denman et al. (38). We performed multiple assays to assess the expansion and maturation of iNK cells. However, despite our efforts, neither of the tested protocols with our aAPC line resulted in significant iNK cell expansion, though it was effective with PB-NK cells. Both iNK cell types were able to completely lyse the irradiated K562-mbIL21 cell line within 48 hours, but no logarithmic expansion occurred. After two weeks of culture under expansion conditions, the iNK cells became apoptotic and anergic, with a maximum expansion of only 2-fold (data not shown). Further optimization of this aAPC line is required. Comparing the expansion capacities, maturation and functional activities of iNK cells following co-culture with aAPC lines would be of particular interest.

## 5 Conclusion

In this study, we demonstrate the *in vitro* functional superiority of lymphoid feeder-based iNK cells over those generated using a feeder-free protocol in our assay models. Transcriptional analysis provides new insights into NK cell differentiation, revealing areas for improvement of the differentiation process, particularly through the activation of the Notch signaling pathway. Our findings also indicate that, despite the clinical appeal of feeder-free differentiation protocols, they require further development in order to produce more mature and functional NK cells suitable for medical purposes.

## Data Availability

The original contributions presented in the study are included in the article/**Supplementary Material**; Transcriptome data were submitted in the following website (https://figshare.com/articles/dataset/RNAseq_transcriptome_matrix_of_human_natural_killer_culture_protocols/27089071?file=49365322) under the accession number DOI : 10.6084/m9.figshare.27089071. Further inquiries can be directed to the corresponding author.

## Supplementary material

Supplementary Figure 1 Characterization of the iPSC line (PB68.6). **(A)** Representative flow cytometry of pluripotent markers of PB68.6 at passage 6 in red, isotype control is represented in blue. **(B)** Karyotype representation of PB68.6 at passage 9. **(C)** Teratoma tissue from PB68.6 showing normal ectodermal (Ne: neural crest and glial tissues, Ma: malpighian epitheliums) endodermal (Ep: Pulmonary epitheliums) and mesodermal (Ca: cartilaginous areas, Bo: Bone structures) differentiation. (magnification 10X).

Supplementary Figure 2 Differentiation kinetics of iNK cells. **(A)** Characterization of hematopoietic and NK markers during differentiation. **(B)** Characterization of CD34^+^CD7^+^CD45RA^+^ lymphoid progenitor during NK differentiation **(C)** Characterization of CD7 and CD56 markers during differentiation. **(D)** Ki67 expression in iNKs at the end point of differentiation after IL2 stimulation for 24 h. Means and SD are represented. P-values were calculated using a two-tailed Student’s t-test. ns, not significant; *P < 0.05; **P < 0.01; ***P < 0.001.

Supplementary Figure 3 Differentially expressed genes between OP9-DLL4 iNK cells and feeder-free iNK cells. **(A)** Volcano plot of differentially expressed genes (DEGs, n=2148) between OP9-DLL4 iNK cells and feeder-free iNK cells. **(B)** Principal component analysis based on expression of the 2148 DEGs. **(C)** Unsupervised clustering (Euclidean distances) with expression heatmap drawn based on the 2148 DEGs.

Supplementary Figure 4 Prediction of transcription factor interactions in the transcriptome. **(A)** Barplot representing enrichment in combinations of transcription factors observed in OP9-DLL4 iNKs. **(B)** Enrichment network for combinations of transcription factors observed in OP9-DLL4 iNKs. **(C)** Barplot representing enrichment in combinations of transcription factors observed in feeder-free iNKs. **(D)** Enrichment network for combinations of transcription factors observed in feeder-free iNK. The line represents the significance of the TF-TF link (Transcription Factor) after enrichment on the TF database: red P-value<=0.05, black P-value>0.05.

Supplementary Figure 5 Proliferation assay using K562-mbIL21. **(A)** Representative flow cytometry of IL21 staining of K562 WT (blue) and K562-mbIL21 (red). **(B)** Characterization of iNK OP9-DLL4 and iNK feeder-free before and after expansion using K562-mbIL21 for 2 weeks using NK expansion media. **(C)** Degranulation assay of iNK OP9-DLL4 and feeder-free iNK cells before and after expansion with K562-mbIL21 after 4 hours of co-culture.
