# Supplementary figures and images for: Comparative analysis of iPSC-derived NK cells from two differentiation strategies reveals distinct signatures and cytotoxic activities
Source: Front Immunol. 2024 Oct 9;15:1463736. doi: 10.3389/fimmu.2024.1463736 (PMC11496199; doi:10.3389/fimmu.2024.1463736)

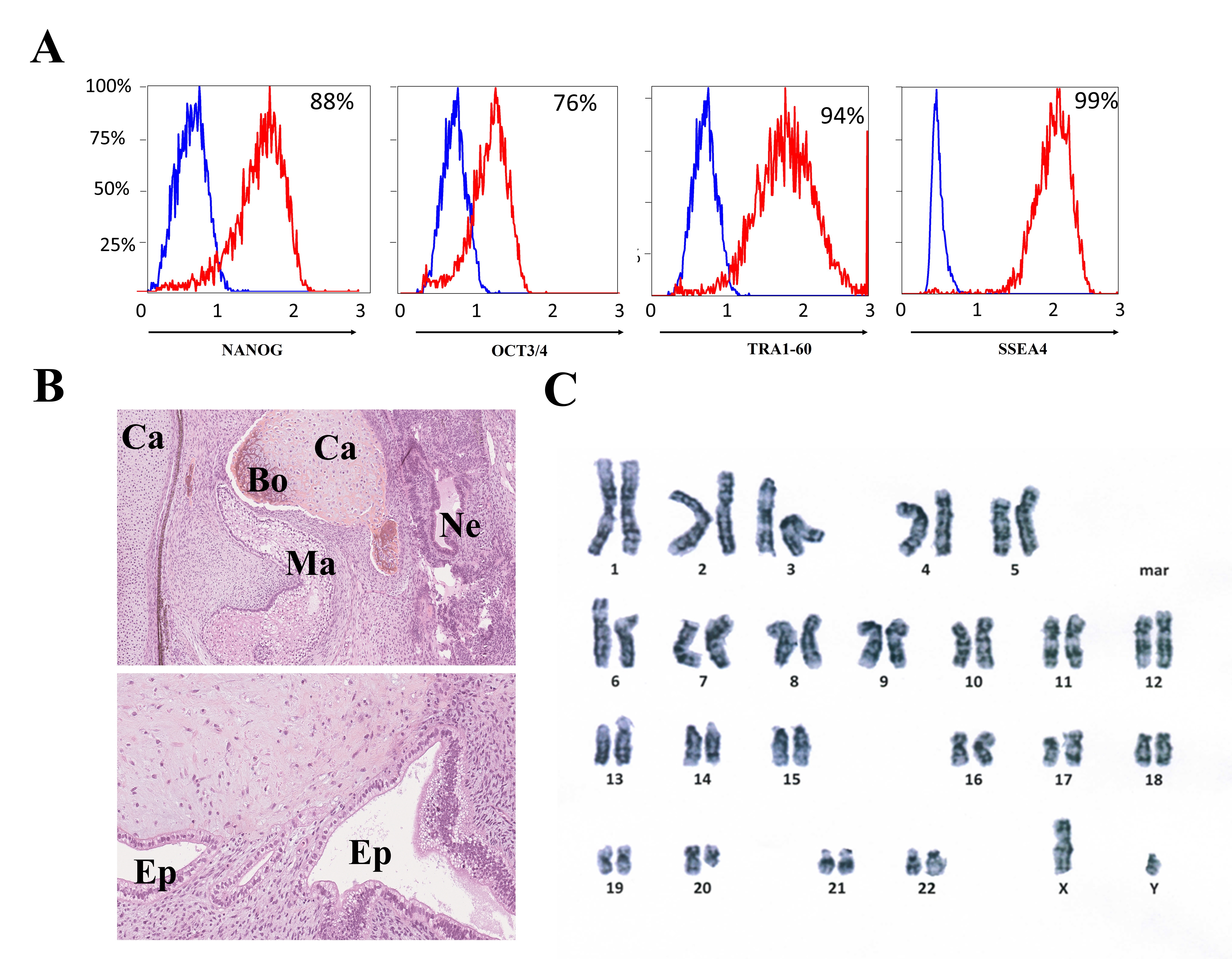

Supplement: Supplementary Figure 1 — Characterization of the iPSC line (PB68.6). (A) Representative flow cytometry of pluripotent markers of PB68.6 at passage 6 in red, isotype control is represented in blue. (B) Karyotype representation of PB68.6 at passage 9. (C) Teratoma tissue from PB68.6 showing normal ectodermal (Ne: neural crest and glial tissues, Ma: malpighian epitheliums) endodermal (Ep: Pulmonary epitheliums) and mesodermal (Ca: cartilaginous areas, Bo: Bone structures) differentiation. (magnification 10X). [file Image1.jpg]

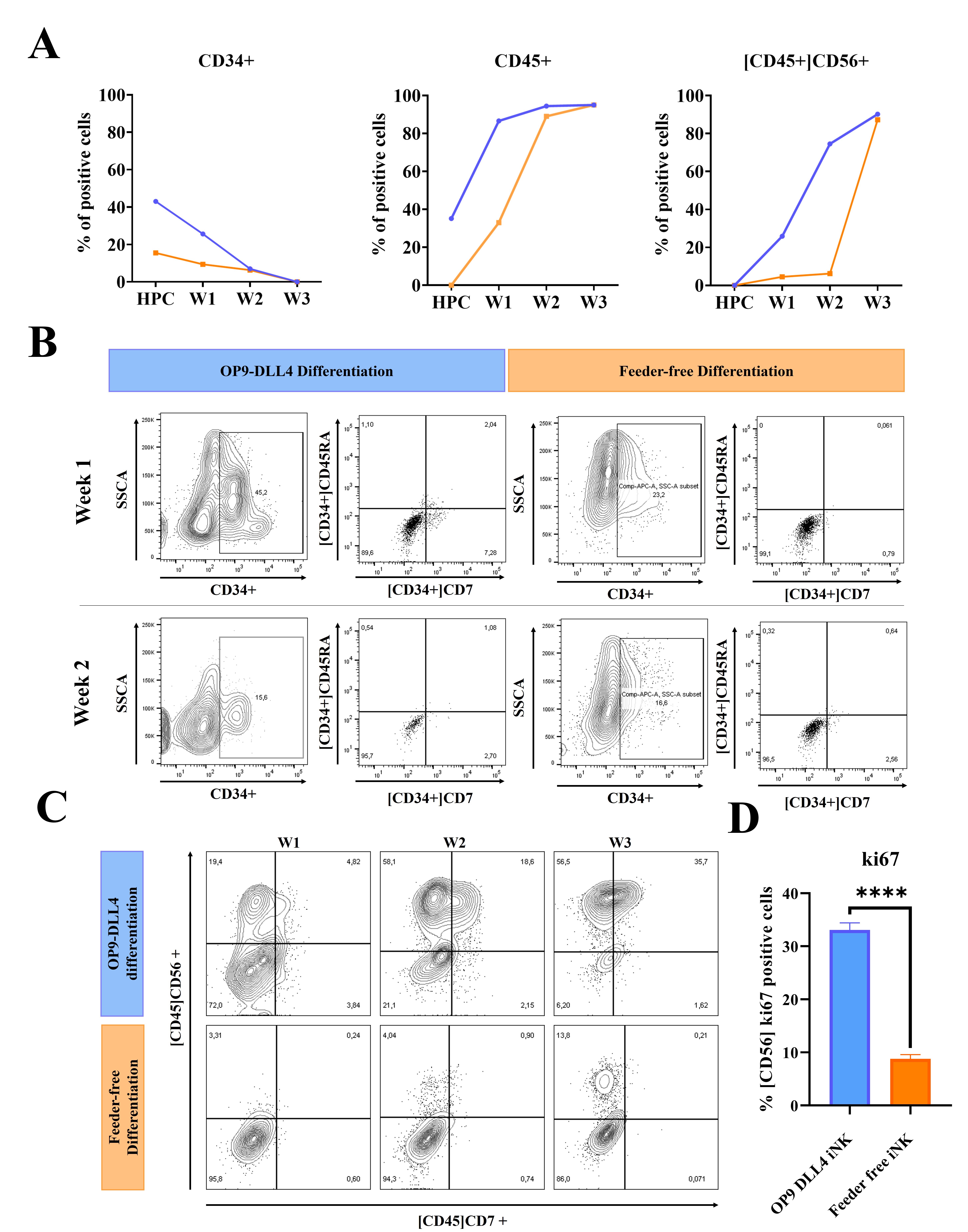

Supplement: Supplementary Figure 2 — Differentiation kinetics of iNK cells. (A) Characterization of hematopoietic and NK markers during differentiation. (B) Characterization of CD34+CD7+CD45RA+ lymphoid progenitor during NK differentiation (C) Characterization of CD7 and CD56 markers during differentiation. (D) Ki67 expression in iNKs at the end point of differentiation after IL2 stimulation for 24 h. Means and SD are represented. P-values were calculated using a two-tailed Student’s t-test. ns, not significant; *P < 0.05; **P < 0.01; ***P < 0.001. [file Image2.jpg]

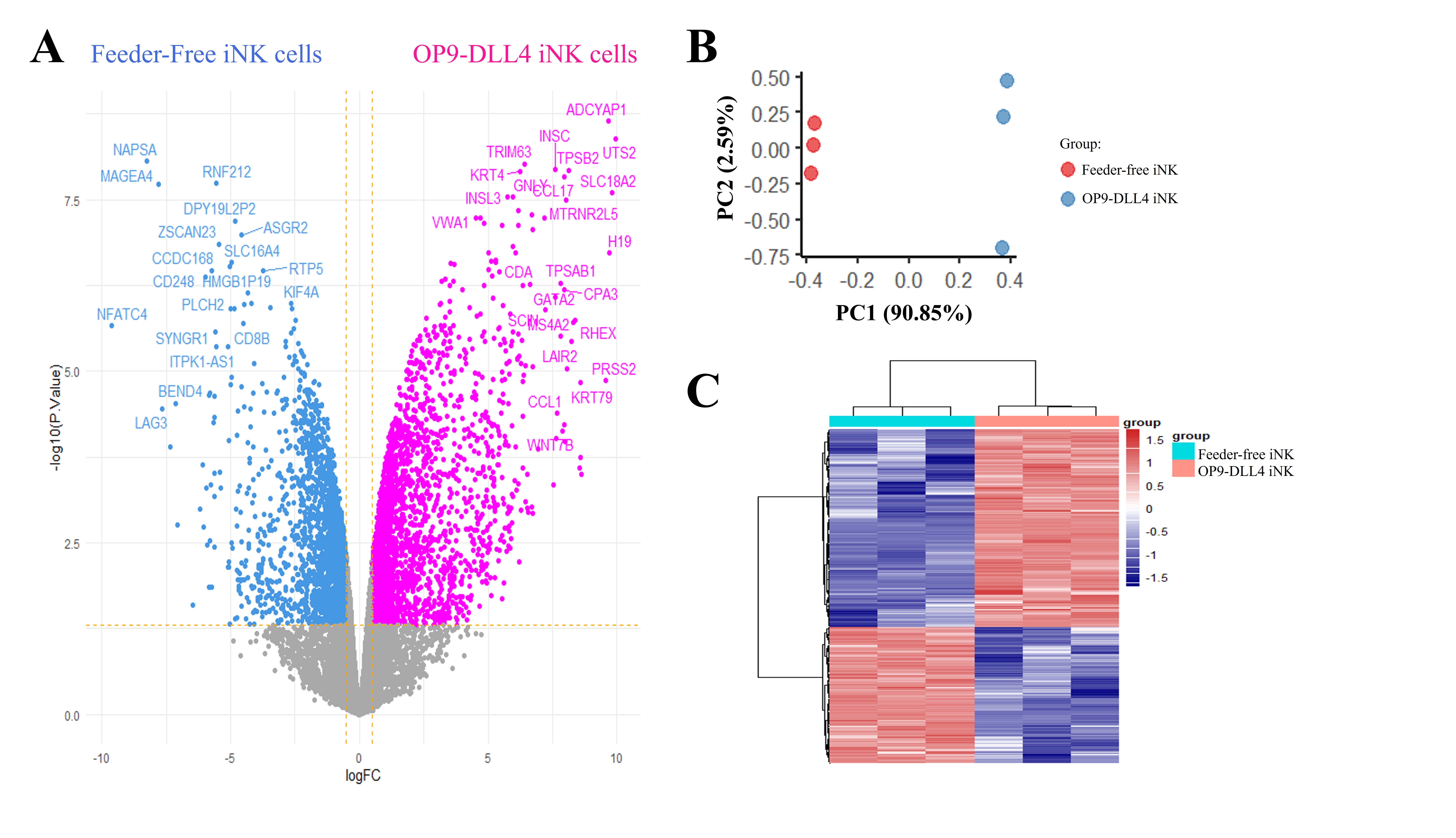

Supplement: Supplementary Figure 3 — Differentially expressed genes between OP9-DLL4 iNK cells and feeder-free iNK cells. (A) Volcano plot of differentially expressed genes (DEGs, n=2148) between OP9-DLL4 iNK cells and feeder-free iNK cells. (B) Principal component analysis based on expression of the 2148 DEGs. (C) Unsupervised clustering (Euclidean distances) with expression heatmap drawn based on the 2148 DEGs. [file Image3.jpg]

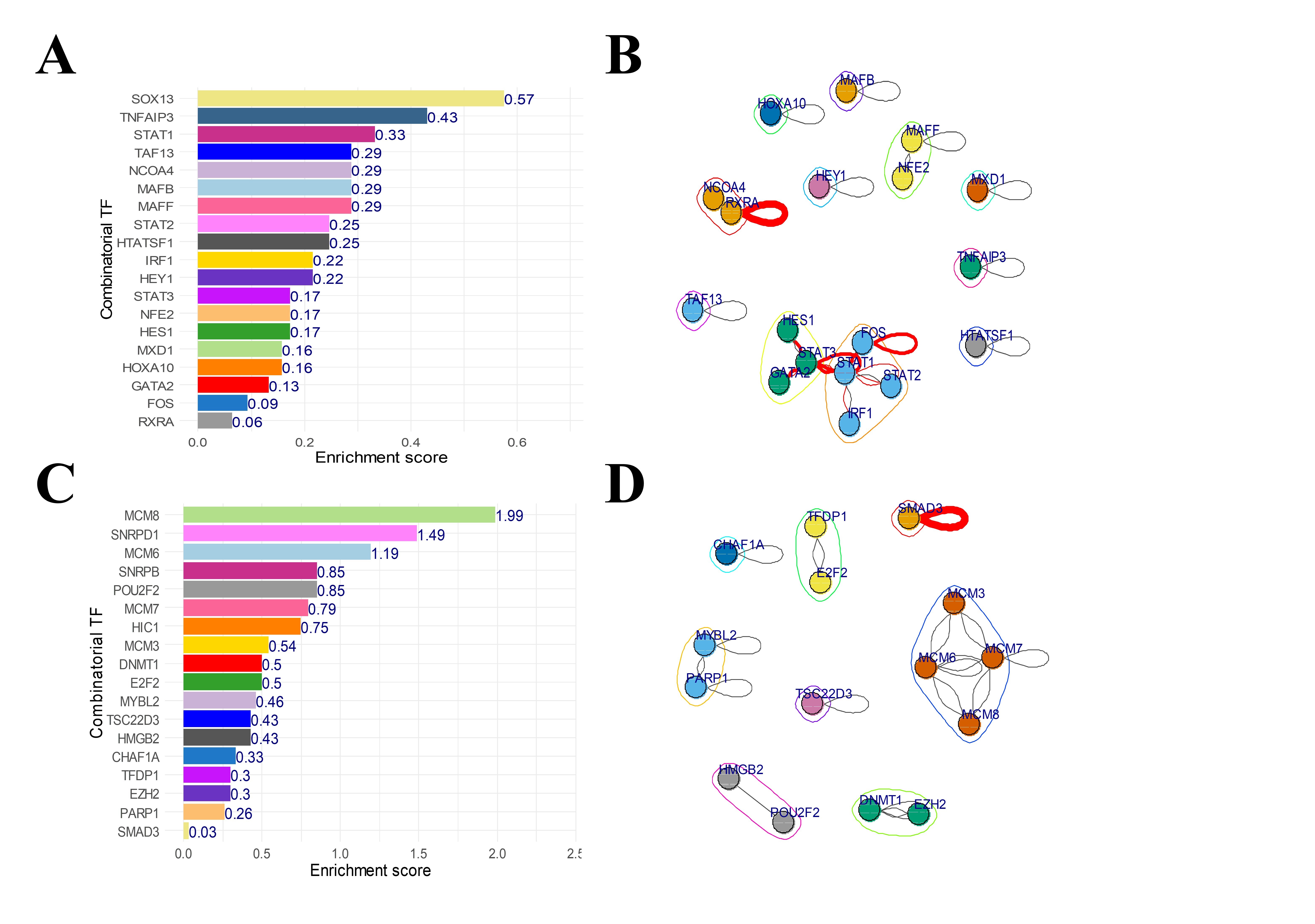

Supplement: Supplementary Figure 4 — Prediction of transcription factor interactions in the transcriptome. (A) Barplot representing enrichment in combinations of transcription factors observed in OP9-DLL4 iNKs. (B) Enrichment network for combinations of transcription factors observed in OP9-DLL4 iNKs. (C) Barplot representing enrichment in combinations of transcription factors observed in feeder-free iNKs. (D) Enrichment network for combinations of transcription factors observed in feeder-free iNK. The line represents the significance of the TF-TF link (Transcription Factor) after enrichment on the TF database: red P-value<=0.05, black P-value>0.05. [file Image4.jpg]

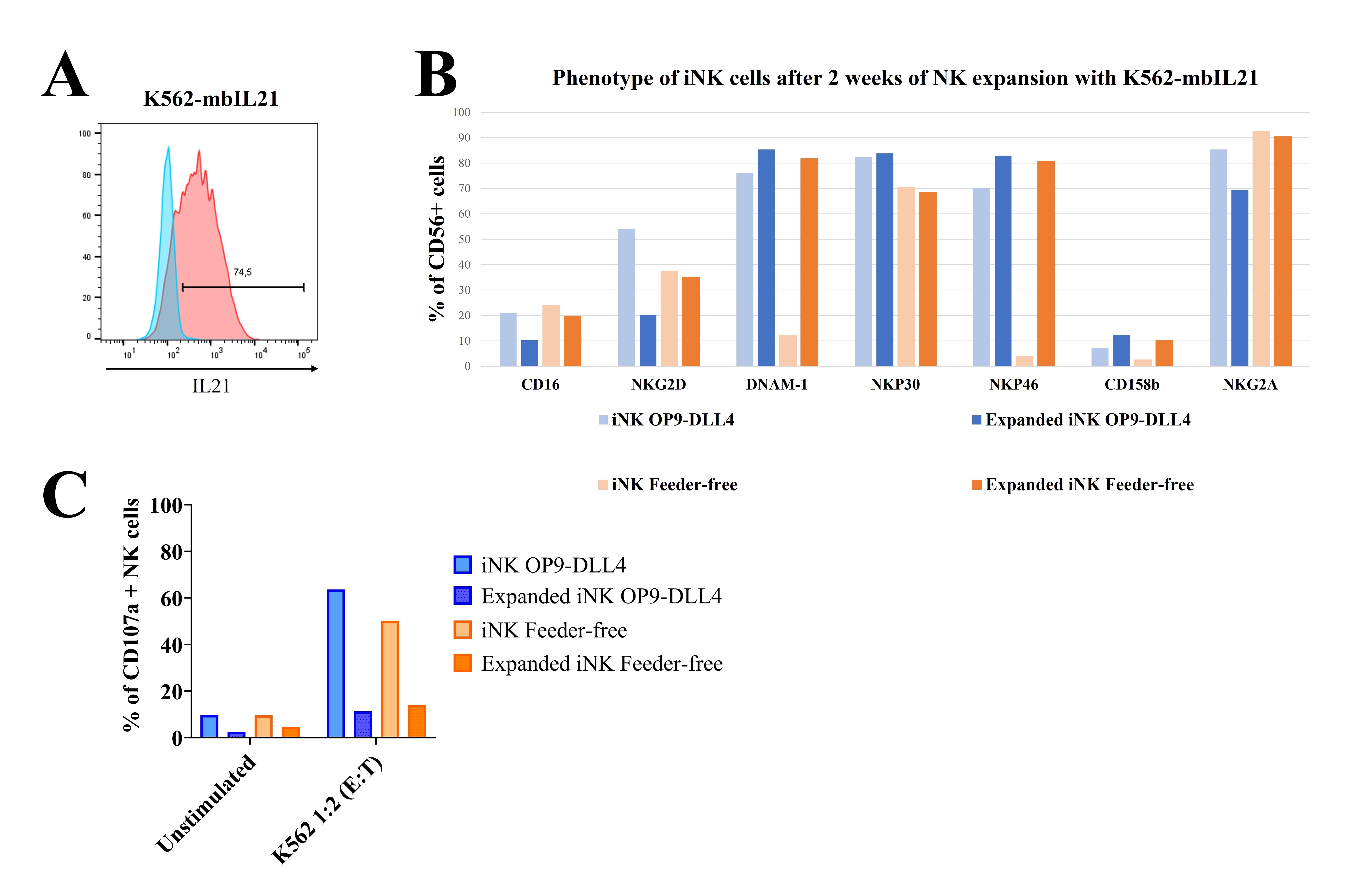

Supplement: Supplementary Figure 5 — Proliferation assay using K562-mbIL21. (A) Representative flow cytometry of IL21 staining of K562 WT (blue) and K562-mbIL21 (red). (B) Characterization of iNK OP9-DLL4 and iNK feeder-free before and after expansion using K562-mbIL21 for 2 weeks using NK expansion media. (C) Degranulation assay of iNK OP9-DLL4 and feeder-free iNK cells before and after expansion with K562-mbIL21 after 4 hours of co-culture. [file Image5.jpg]
