# Supplementary material for: Comparative analysis of iPSC-derived NK cells from two differentiation strategies reveals distinct signatures and cytotoxic activities
Source: Front Immunol. 2024 Oct 9;15:1463736. doi: 10.3389/fimmu.2024.1463736 (PMC11496199; doi:10.3389/fimmu.2024.1463736)
Supplement: Supplementary file 6 [file Table1.docx]

**Supplemental Tables**

**Supplemental Table 1 : Antibodies used for FACS and their respective references**

| **Antibody and Clone** | **Reference** |
| --- | --- |
| CD56-VioBrightV423, REA196 | 130-126-299 Miltenyi Biotec |
| CD314 (NKG2D)-APC, REA797 | 130-111-846 Miltenyi Biotec |
| CD335 (NKp46)-VioBright 515, REA808 | 130-112-120 Miltenyi Biotec |
| CD337 (NKp30)-PE, REA823 | 130-112-430 Miltenyi Biotec |
| CD226 (DNAM-1)-PE-Vio770, REA1040 | 130-117-492 Miltenyi Biotec |
| CD3-PerCP-Vio700, REA613 | 130-113-141 Miltenyi Biotec |
| CD16-APC-Vio770, REA423 | 130-113-390 Miltenyi Biotec |
| CD159a (NKG2A)-PE-Vio770, REA110 | 130-113-567 Miltenyi Biotec |
| TIGIT-VioBright 515 REA1004 | 130-116-818 Miltenyi Biotec |
| CD158b (KIR2DL2/DL3)-PE, REA1006 | 130-116-832 Miltenyi Biotec |
| CD45-PE, mouse IgG2a, 5B1 | 170-081-061 Miltenyi Biotec |
| CD34-APC, mouse IgG2aκ, AC136 | 130-113-176 Miltenyi Biotec |
| CD107a-PE, REA792 | 130-111-621 Miltenyi Biotec |
| NK80-PE, REA845 | 130-112-590 Miltenyi Biotec |
| KI67-APC, REA183 | 130-120-416 Miltenyi Biotec |
| NANOG-APC, REA314 | 130-120-704 Miltenyi Biotec |
| TRA1-60-PE, REA157 | 130-122-921 Miltenyi Biotec |
| SSEA4-Viogreen, REA101 | 130-123-901 Miltenyi Biotec |
| OCT34-PE, REA338 | 130-123-771 Miltenyi Biotec |
| IL21-FITC, REA1039 | 130-130-441 Miltenyi Biotec |

**Supplemental Table 2 : Table of differential expressed genes between OP9DLL4 iNK and Feeder-free iNK (n=2148).**

**Supplemental Table 3: Statistics table of the 164 human transcription factors regulated between iNKs (OP9-DLL4 and Feeder-free) and human primary NK.**

| **gene** | **Fisher statistics** | **P-Value** | **Adjusted P-Value** |
| --- | --- | --- | --- |
| HOXB5 | 389.634 | 1.09E-08 | 3.80E-06 |
| HOXB6 | 280.260 | 3.99E-08 | 6.98E-06 |
| NFATC4 | 202.445 | 1.43E-07 | 1.23E-05 |
| NR2F6 | 194.812 | 1.66E-07 | 1.23E-05 |
| LIN28B | 192.120 | 1.76E-07 | 1.23E-05 |
| HOXA10 | 140.538 | 5.94E-07 | 3.47E-05 |
| BATF2 | 127.539 | 8.66E-07 | 4.33E-05 |
| LHX2 | 119.280 | 1.12E-06 | 4.36E-05 |
| BHLHE41 | 118.682 | 1.14E-06 | 4.36E-05 |
| EGR3 | 116.132 | 1.24E-06 | 4.36E-05 |
| ARNTL2 | 111.502 | 1.46E-06 | 4.60E-05 |
| ZNF470 | 107.597 | 1.67E-06 | 4.60E-05 |
| ZNF165 | 105.717 | 1.79E-06 | 4.60E-05 |
| NR4A2 | 104.944 | 1.84E-06 | 4.60E-05 |
| ZSCAN23 | 98.152 | 2.38E-06 | 5.55E-05 |
| ZNF331 | 94.324 | 2.77E-06 | 5.92E-05 |
| IRX3 | 93.002 | 2.92E-06 | 5.92E-05 |
| GATA2 | 91.109 | 3.16E-06 | 5.92E-05 |
| JUN | 90.730 | 3.22E-06 | 5.92E-05 |
| FOS | 89.534 | 3.38E-06 | 5.92E-05 |
| THAP8 | 87.091 | 3.76E-06 | 6.08E-05 |
| HEY1 | 85.926 | 3.96E-06 | 6.08E-05 |
| ETV4 | 85.061 | 4.11E-06 | 6.08E-05 |
| ZBTB21 | 84.753 | 4.17E-06 | 6.08E-05 |
| ZEB2 | 78.471 | 5.59E-06 | 7.83E-05 |
| ZNF2 | 71.067 | 8.14E-06 | 1.10E-04 |
| ZNF695 | 69.896 | 8.67E-06 | 1.12E-04 |
| HLF | 68.690 | 9.26E-06 | 1.16E-04 |
| ZNF697 | 66.802 | 1.03E-05 | 1.21E-04 |
| POU2F1 | 66.664 | 1.04E-05 | 1.21E-04 |
| KLF3 | 65.442 | 1.11E-05 | 1.21E-04 |
| CSRNP1 | 65.384 | 1.12E-05 | 1.21E-04 |
| CASZ1 | 65.021 | 1.14E-05 | 1.21E-04 |
| FOXB1 | 64.432 | 1.18E-05 | 1.21E-04 |
| ZNF683 | 62.138 | 1.35E-05 | 1.35E-04 |
| HES4 | 59.890 | 1.55E-05 | 1.48E-04 |
| KLF9 | 59.742 | 1.57E-05 | 1.48E-04 |
| ZNF844 | 55.419 | 2.07E-05 | 1.91E-04 |
| MAFF | 53.751 | 2.32E-05 | 2.08E-04 |
| CXXC4 | 52.790 | 2.48E-05 | 2.17E-04 |
| ZNF175 | 51.536 | 2.71E-05 | 2.32E-04 |
| HOXA5 | 50.846 | 2.85E-05 | 2.37E-04 |
| NR1D2 | 50.557 | 2.91E-05 | 2.37E-04 |
| ZBTB10 | 48.519 | 3.39E-05 | 2.70E-04 |
| KLF2 | 45.932 | 4.15E-05 | 3.23E-04 |
| ZNF334 | 45.333 | 4.36E-05 | 3.32E-04 |
| ZMAT1 | 43.441 | 5.09E-05 | 3.79E-04 |
| FOSB | 42.265 | 5.63E-05 | 4.11E-04 |
| SOX13 | 41.856 | 5.84E-05 | 4.17E-04 |
| HIC1 | 41.127 | 6.22E-05 | 4.20E-04 |
| ETV3 | 41.089 | 6.24E-05 | 4.20E-04 |
| TCF7 | 40.974 | 6.31E-05 | 4.20E-04 |
| PRDM1 | 40.866 | 6.37E-05 | 4.20E-04 |
| ZNF19 | 40.184 | 6.77E-05 | 4.36E-04 |
| BHLHE40 | 40.054 | 6.85E-05 | 4.36E-04 |
| CREB3L1 | 39.104 | 7.47E-05 | 4.67E-04 |
| ZNF395 | 38.740 | 7.73E-05 | 4.68E-04 |
| ZKSCAN7 | 38.708 | 7.75E-05 | 4.68E-04 |
| SP140 | 36.773 | 9.33E-05 | 5.54E-04 |
| RORA | 36.533 | 9.55E-05 | 5.57E-04 |
| NR4A3 | 35.233 | 1.09E-04 | 6.24E-04 |
| ZNF418 | 34.604 | 1.16E-04 | 6.55E-04 |
| NFKB2 | 33.931 | 1.25E-04 | 6.85E-04 |
| MXD4 | 33.883 | 1.25E-04 | 6.85E-04 |
| MYBL2 | 33.703 | 1.28E-04 | 6.86E-04 |
| ETV1 | 33.572 | 1.29E-04 | 6.86E-04 |
| ZNF215 | 32.039 | 1.53E-04 | 7.98E-04 |
| IRF1 | 31.689 | 1.59E-04 | 8.18E-04 |
| E2F2 | 31.503 | 1.62E-04 | 8.23E-04 |
| STAT1 | 31.018 | 1.71E-04 | 8.57E-04 |
| HBP1 | 29.779 | 1.98E-04 | 9.76E-04 |
| ZNF350 | 29.051 | 2.16E-04 | 1.05E-03 |
| ZNF618 | 26.114 | 3.13E-04 | 1.50E-03 |
| ZNF517 | 26.039 | 3.16E-04 | 1.50E-03 |
| ZNF485 | 25.942 | 3.20E-04 | 1.50E-03 |
| ARID3A | 25.823 | 3.26E-04 | 1.50E-03 |
| L3MBTL4 | 24.290 | 4.02E-04 | 1.83E-03 |
| VDR | 23.337 | 4.61E-04 | 2.05E-03 |
| ATF3 | 23.296 | 4.64E-04 | 2.05E-03 |
| FIZ1 | 23.074 | 4.79E-04 | 2.10E-03 |
| ZNF804A | 22.526 | 5.20E-04 | 2.25E-03 |
| ZNF93 | 21.901 | 5.72E-04 | 2.44E-03 |
| THRA | 21.701 | 5.90E-04 | 2.49E-03 |
| CBX2 | 21.316 | 6.26E-04 | 2.61E-03 |
| HES1 | 20.662 | 6.96E-04 | 2.86E-03 |
| KLF8 | 20.474 | 7.17E-04 | 2.92E-03 |
| NFE2 | 19.903 | 7.88E-04 | 3.17E-03 |
| ZHX3 | 19.044 | 9.12E-04 | 3.63E-03 |
| IRX5 | 18.452 | 1.01E-03 | 3.98E-03 |
| HOXB4 | 18.321 | 1.04E-03 | 4.01E-03 |
| IRX2 | 18.285 | 1.04E-03 | 4.01E-03 |
| PRR12 | 17.588 | 1.18E-03 | 4.46E-03 |
| E2F1 | 17.578 | 1.19E-03 | 4.46E-03 |
| SALL4 | 16.699 | 1.40E-03 | 5.22E-03 |
| NR1H3 | 16.235 | 1.53E-03 | 5.65E-03 |
| BNC2 | 16.063 | 1.59E-03 | 5.77E-03 |
| BCL6 | 15.981 | 1.61E-03 | 5.77E-03 |
| ARNT2 | 15.970 | 1.62E-03 | 5.77E-03 |
| ZNF775 | 15.874 | 1.65E-03 | 5.83E-03 |
| NR1D1 | 15.697 | 1.71E-03 | 5.98E-03 |
| ETV7 | 15.631 | 1.73E-03 | 6.00E-03 |
| BATF3 | 15.353 | 1.83E-03 | 6.29E-03 |
| FOXD1 | 15.239 | 1.88E-03 | 6.37E-03 |
| EBF4 | 15.176 | 1.90E-03 | 6.40E-03 |
| E2F8 | 14.975 | 1.98E-03 | 6.61E-03 |
| ZNF233 | 14.473 | 2.21E-03 | 7.29E-03 |
| ZBTB20 | 14.127 | 2.38E-03 | 7.79E-03 |
| PLAG1 | 13.995 | 2.45E-03 | 7.94E-03 |
| CENPA | 13.862 | 2.52E-03 | 8.11E-03 |
| ZNF429 | 13.190 | 2.94E-03 | 9.36E-03 |
| HES6 | 13.040 | 3.05E-03 | 9.61E-03 |
| ZNF487 | 12.896 | 3.15E-03 | 9.85E-03 |
| IKZF5 | 12.525 | 3.45E-03 | 1.07E-02 |
| PHF1 | 12.343 | 3.60E-03 | 1.11E-02 |
| LHX1 | 11.871 | 4.05E-03 | 1.23E-02 |
| THAP2 | 11.838 | 4.08E-03 | 1.23E-02 |
| ZNF333 | 11.789 | 4.13E-03 | 1.24E-02 |
| GTF2IRD1 | 11.714 | 4.21E-03 | 1.25E-02 |
| ZNF829 | 11.633 | 4.30E-03 | 1.26E-02 |
| ZNF503 | 11.623 | 4.31E-03 | 1.26E-02 |
| CREB5 | 11.207 | 4.80E-03 | 1.39E-02 |
| ZNF439 | 10.889 | 5.22E-03 | 1.50E-02 |
| ZNF443 | 10.711 | 5.48E-03 | 1.56E-02 |
| ZBTB32 | 10.572 | 5.69E-03 | 1.61E-02 |
| LEF1 | 10.437 | 5.91E-03 | 1.65E-02 |
| IKZF4 | 9.941 | 6.80E-03 | 1.89E-02 |
| ZNF595 | 9.892 | 6.89E-03 | 1.90E-02 |
| ZNF133 | 9.695 | 7.30E-03 | 2.00E-02 |
| ZNF140 | 9.550 | 7.62E-03 | 2.07E-02 |
| ZNF615 | 9.416 | 7.92E-03 | 2.13E-02 |
| ZNF768 | 9.328 | 8.13E-03 | 2.17E-02 |
| ZNF528 | 9.153 | 8.58E-03 | 2.27E-02 |
| ZNF571 | 9.014 | 8.95E-03 | 2.35E-02 |
| TFCP2L1 | 8.946 | 9.14E-03 | 2.37E-02 |
| FOXP4 | 8.944 | 9.14E-03 | 2.37E-02 |
| TIGD1 | 8.553 | 1.03E-02 | 2.65E-02 |
| TAL1 | 8.522 | 1.04E-02 | 2.65E-02 |
| ZFP92 | 8.503 | 1.05E-02 | 2.65E-02 |
| BCL11A | 8.493 | 1.05E-02 | 2.65E-02 |
| NEUROD2 | 8.456 | 1.07E-02 | 2.67E-02 |
| FOSL1 | 8.147 | 1.18E-02 | 2.92E-02 |
| ZNF831 | 8.133 | 1.18E-02 | 2.92E-02 |
| ZNF285 | 7.713 | 1.36E-02 | 3.34E-02 |
| AEBP1 | 7.456 | 1.49E-02 | 3.62E-02 |
| ZNF613 | 7.430 | 1.50E-02 | 3.63E-02 |
| HDX | 7.409 | 1.51E-02 | 3.63E-02 |
| ZNF737 | 7.352 | 1.54E-02 | 3.68E-02 |
| ZBTB3 | 7.292 | 1.58E-02 | 3.73E-02 |
| PRDM13 | 7.250 | 1.60E-02 | 3.76E-02 |
| SOX6 | 7.208 | 1.63E-02 | 3.79E-02 |
| MSC | 7.183 | 1.64E-02 | 3.80E-02 |
| GLI4 | 7.033 | 1.73E-02 | 3.99E-02 |
| ZNF823 | 6.996 | 1.75E-02 | 4.00E-02 |
| ZNF280B | 6.973 | 1.77E-02 | 4.00E-02 |
| ZNF660 | 6.959 | 1.78E-02 | 4.00E-02 |
| MEIS2 | 6.954 | 1.78E-02 | 4.00E-02 |
| ZBTB9 | 6.642 | 2.00E-02 | 4.46E-02 |
| ZNF155 | 6.552 | 2.07E-02 | 4.58E-02 |
| ZNF232 | 6.514 | 2.10E-02 | 4.60E-02 |
| MYCN | 6.502 | 2.11E-02 | 4.60E-02 |
| ZFHX2 | 6.494 | 2.11E-02 | 4.60E-02 |
| PROX2 | 6.371 | 2.22E-02 | 4.77E-02 |
| MYC | 6.364 | 2.22E-02 | 4.77E-02 |
| PLSCR1 | 6.248 | 2.32E-02 | 4.96E-02 |
